# Jatrophane Diterpenoids from the Seeds of *Euphorbia peplus* with Potential Bioactivities in Lysosomal-Autophagy Pathway

**DOI:** 10.1007/s13659-021-00301-4

**Published:** 2021-03-14

**Authors:** Yan-Ni Chen, Xiao Ding, Dong-Mei Li, Qing-Yun Lu, Shuai Liu, Ying-Yao Li, Ying-Tong Di, Xin Fang, Xiao-Jiang Hao

**Affiliations:** 1 State Key Laboratory of Phytochemistry and Plant Resources in West China, Kunming Institute of Botany, Chinese Academy of Sciences, Kunming, People’s Republic of China; 2 University of Chinese Academy of Sciences, Beijing, 100049 People’s Republic of China; 3 Yunnan University, Kunming, People’s Republic of China

**Keywords:** *Euphorbia peplus*, Jatrophane, Lysosomal biogenesis activity, Macrocyclic diterpenoid

## Abstract

**Abstract:**

Euphopepluanones F − K (**1** − **4**), four new jatrophane type diterpenoids were isolated from the seeds of *Euphorbia peplus*, along with eight known diterpenoids (**5** − **12**). Their structures were established on the basis of extensive spectroscopic analysis and X-ray crystallographic experiments. The new compounds **1** − **4** were assessed for their activities to induce lysosomal biogenesis through LysoTracker Red staining. Compound **2** significantly induced lysosomal biogenesis. In addition, compound **2** could increase the number of LC3 dots, indicating that it could activate the lysosomal-autophagy pathway.

**Graphic Abstract:**

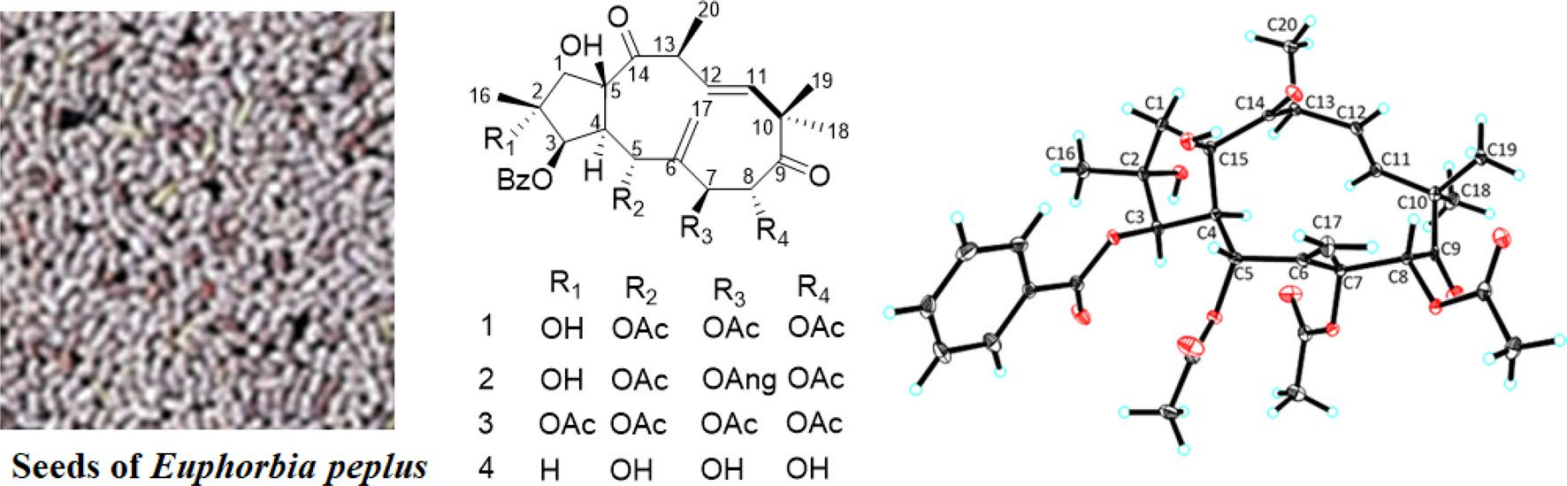

**Supplementary Information:**

The online version contains supplementary material available at 10.1007/s13659-021-00301-4.

## Introduction

Plants of the genus *Euphorbia* are used in traditional medicines for treatments of digestive system disorders, skin ailments, infections, inflammation, and injuries world-wide [1]. The medicinal usage of *Euphorbia* plants is attributed to the structurally diverse polycyclic diterpenoids produced by these plants, which have reported more than 20 skeletal types [2, 3]. Euphodendroidin D, for example, is a jatrophane type diterpenoid from *E. dendroides* that show inhibition of the transport activity of P-glycoprotein, an ABC transporter protein related to multidrug resistance by decreasing the intracellular concentration of drugs [4]. Resiniferatoxin, a daphnane diterpenoid from *E. resinifera*, is known for the alleviation of neuropathic pain and is in phase I human clinical trials in treating severe pain in cancer [5]. In 2012, an ingenane type diterpenoid ingenol 3-angelate from *E. peplus* was approved by FDA for the treatment of actinic keratosis, a precancerous skin condition [6, 7].

**Fig. 1:**
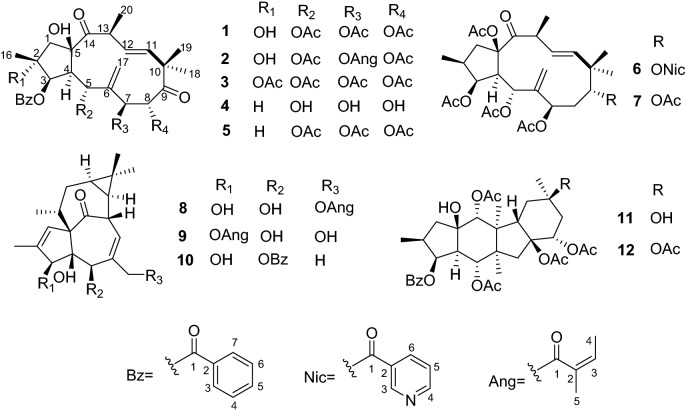
Chemical structures of compounds **1**−**12**

*E. peplus* Linn., a small annual weed native to Mediterranean coast, was introduced into Yunan province of China [8]. The sap from *E. peplus* has been used in folk medicine for the treatment of asthma, catarrh and internal tumors [9]. Recently, our group discovered ingenane type diterpenoids 20-deoxyingenol and its analogues from *E. peplus*, possessing activity to promote lysosome biogenesis, limit amyloid plaque formation in APP/SP1 mice’s brain, which suggested that the potential of these compounds for the treatment of Alzheimer disease [10]. The subsequent phytochemical studies of the plants of the genus *Euphorbia* led to isolation of several novel diterpenoids with significant bioactivities [11–16]. In our continuing efforts to uncover structurally novel diterpenes capable of inducing lysosomal biogenesis*,* four new jatrophane type diterpenoids euphopepluanones F − K (**1** − **4**), along with eight known diterpenoids (**5** − **12**) were obtained from the seed of *E. peplus* (Fig. 1). Their structures were elucidated based on extensive NMR, X-ray crystallographic and electronic circular dichroism (ECD) experiments. Furthermore, the activity of compounds **1** − **4** in inducing lysosomal biogenesis were tested, in which only **2** displayed significant activity. Herein, we reported the structural elucidation and biological evaluation of these compounds.

## Results and Discussion

### Structure Elucidation

**Fig. 2:**
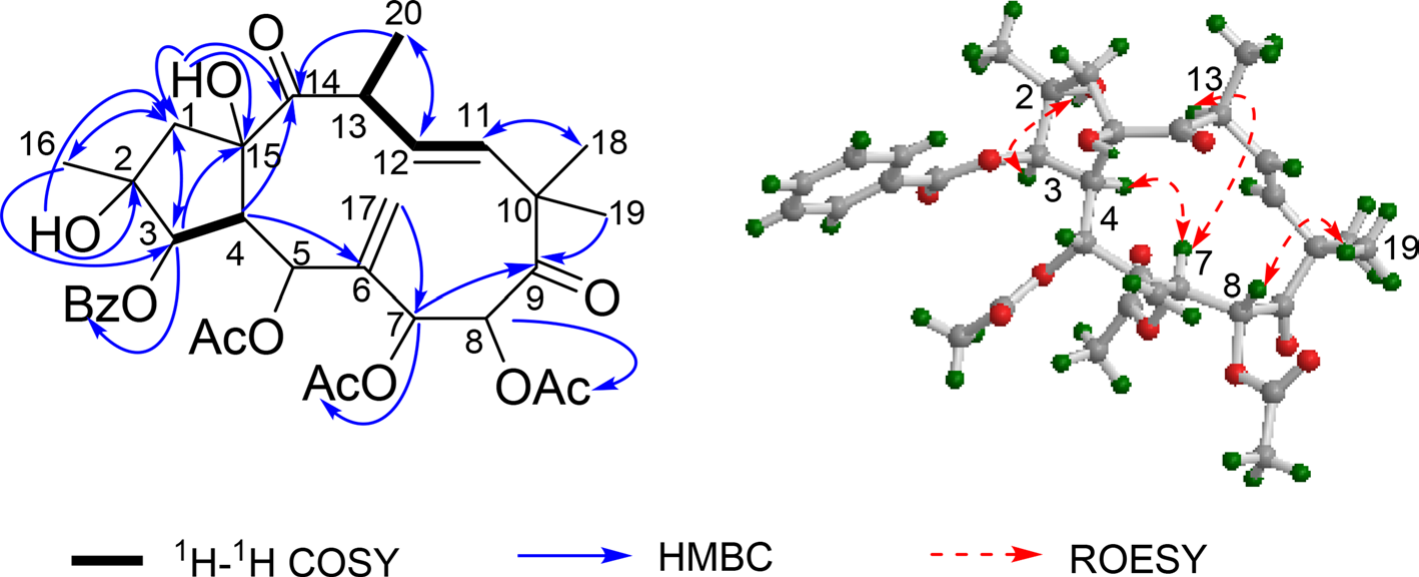
Key ^1^H- ^1^H COSY, HMBC, and ROESY correlations of compound **1**

Euphopepluanone F (**1**), was obtained as colorless crystals. The molecular formula of **1** was established as C_33_H_40_O_12_ based on its positive HRESIMS ([M + Na]^+^, *m/z* 651.2426, calcd for C_33_H_40_O_12_Na, 651.2412). Its IR spectrum indicated the absorption bands for hydroxyl (3481 cm^−1^), carbonyl of ester (1743 cm^−1^), ketone (1723 cm^−1^), and double bond (1650 cm^−1^). Its 1D NMR spectra (Table 1) showed typical signals for three acetoxy groups (*δ*_C_ 170.4, 20.5; 170.0, 20.8; and 168.9, 20.7; *δ*_H_ 2.08, 2.05, and 1.67), and a benzoyloxy group (*δ*_C_ 165.6; *δ*_H_ 8.12, 7.57 and 7.45). In addition to these signals, the ^13^C NMR spectrum displayed four methyls (*δ*_C_ 20.7, 23.1, 24.0 and 25.2), one *sp*^3^ methylene (*δ*_C_ 52.3), one *sp*^2^ methylene (*δ*_C_ 114.2), five *sp*^3^ methines (*δ*_C_ 42.8, 47.7, 65.2, 72.7 and 81.0), two *sp*^2^ methines (*δ*_C_ 133.1 and 136.1), three *sp*^3^ quaternary carbons (*δ*_C_ 49.6, 79.1 and 85.2) and three *sp*^2^ quaternary carbons (*δ*_C_ 138.0, 204.9 and 213.2). These signals account for 19 carbons, indicating the absence of one carbon signal that may be attributed to conformational exchange of the jatrophane type diterpenoid molecule [17, 18]. The spectroscopic data of **1** was similar to (2S*,3S*,4R*,5R*,7S*,8R*,13S*,15R*)-5,7,8-triacetoxy-3-benzoyloxy-15-hydroxyjatropha-6(17),11*E*-diene-9,14-dione (**5**) [17] except for the presence of signals responsible for an hydroxy group (*δ*_H_ 2.33) and an additional oxygenated quaternary carbon (*δ*_C_ 79.1), the absence of the proton signal at C-2. These differences suggested that compound **1** harbors an additional hydroxy group attached at C-2, which was confirmed by the HMBC correlations from the hydroxy group to C-1 and C-2 (Fig. 2).

**Table 1:** ^1^H (500 MHz) and ^13^C (125 MHz) NMR Data of **1** − **4** in CDCl_3_, (*J* in Hz, *δ* in ppm)

No.	1		2		3		4	
	*δ*_H_	*δ*_C_	*δ*_H_	*δ*_C_	*δ*_H_	*δ*_C_	*δ*_H_	*δ*_C_
1a	2.47, d (15.4)	52.3, t	2.48, d (15.4)	52.3, t	3.17, d (16.4)	49.7, t	2.48, dd (14.4, 8.5)	46.2, t
1b	2.23, d (15.4)		2.24, d (15.4)		2.29, d (16.4)		2.02, dd (14.4, 12.0)	
2	–	79.1, s	–	79.3, s	–	88.4, s	2.34, m	38.9, d
3	5.54, d (3.7)	81.0, d	5.56, d (3.8)	81.3, d	5.92, d (3.8)	78.1, d	5.82, br. s	78.3, d
4	3.54, dd (7.8, 3.7)	47.7, d	3.60, dd (8.3,3.8)	47.6, d	3.28, br. s	47.8, d	2.58, br. d (6.3)	53.4, d
5	ND	ND	ND	ND	ND	ND	4.42, brs	72.4, d
6	–	138.0, s	–	138.3, s	–	ND	–	144.5, s
7	5.96, d (4.6)	65.2, d	6.09, br. s	64.3, d	5.96, d (5.5)	65.1, d	4.27, br. s	65.6, d
8	5.38, br. s	72.7, d	5.41, br. s	73.0, d	5.36, br. s	72.5, d	4.54, br. s	71.5, d
9	–	204.9, s	–	204.6, s	–	204.5, s	–	211.1, s
10	–	49.6, s	–	49.5, s	–	49.9, s	–	48.5, s
11	5.99, d (16.0)	136.1, d	6.06, d (16.0)	136.2, d	5.96, d (16.0)	136.5, d	5.73, d (15.9)	136.1, d
12	5.81, dd (16.0, 9.8)	133.1, d	5.82, dd (16.0,9.9)	132.9, d	5.82, dd (16.0, 9.6)	132.2, d	5.66, dd (15.9,9.3)	131.8, d
13	4.38, dd (9.8, 6.6)	42.8, d	4.39, m	42.8, d	3.78, m	43.8, d	3.54, m	43.8, d
14	–	213.2, s	─	213.3, s	–	212.1, s	–	211.9, s
15	–	85.2, s	─	85.1, s	–	85.5, s	–	85.9, s
16	1.38, s	23.1, q	1.39, s	23.3, q	1.61, s	19.0, q	1.13, d (6.3)	14.0, q
17a	5.32, br. s	114.2, t	5.33, br. s	ND	ND	ND	5.34, br. s	ND
17b	5.44, br. s		–		ND		5.45, br. s	
18	1.22, s	24.0, q	1.24, s	24.0, q	1.22, s	23.8, q	1.31, s	23.8, q
19	1.33, s	25.2, q	1.34, s	25.2, q	1.34, s	25.0, q	1.26, s	25.4, q
20	1.35, d (6.6)	20.7, q	1.38, d (6.6)	20.7, q	1.37, d (6.7)	20.6, q	1.40, d (6.5)	20.5, q
2-OH	2.33, s	–	ND	–				
5-OH							ND	–
7-OH							ND	–
8-OH							3.48, s	–
15-OH	4.30, s	–	4.32, s	–	4.31, s		ND	–
2-OAc					–	169.8, s		
					2.19, s	22.3, q		
3-OBz (-OAc)	–	165.6, s	–	165.6, s	–	165.2, s		166.8, s
1ˊ	–	129.6, s	–	129.6, s	–	129.3, s		129.8, s
2ˊ, 6ˊ	8.12, d (7.6)	130.0, d	8.12, d (7.5)	130.0, d	8.14, d (7.6)	130.1, d	8.15, d (7.5)	130.0, d
3ˊ, 5ˊ	7.45, t (7.6)	128.6, d	7.46, t (7.5)	128.5, d	7.46, t (7.6)	128.6, d	7.48, t (7.5)	128.6, d
4ˊ	7.57, t (7.6)	133.4, d	7.57, t (7.5)	133.4, d	7.58, t (7.6)	133.5, d	7.60, t (7.5)	133.3, d
5-OAc	–	168.9, s	–	169.1, s	–	168.9, s		
	1.67, s	20.7, q	1.61, s	20.5, q	1.61, s	20.7, q		
7-OAc	–	170.4, s			–	170.3, s		
	2.08, s	20.5, q			2.06, s	20.4, q		
7-OAng (-OMeBu)			–	166.9, s				
1ˊ			–	127.0, s				
2ˊ			6.16 q (7.3)	140.4, d				
3ˊ			2.01 dd (7.3,1.5)	15.9, q				
4ˊ			1.87 s	20.4, q				
8-OAc	–	170.0, s	–	169.9, s	–	170.0, s		
	2.05, s	20.8, q	2.06 s	20.4, q	2.07, s	20.7, q

**Fig. 3:**
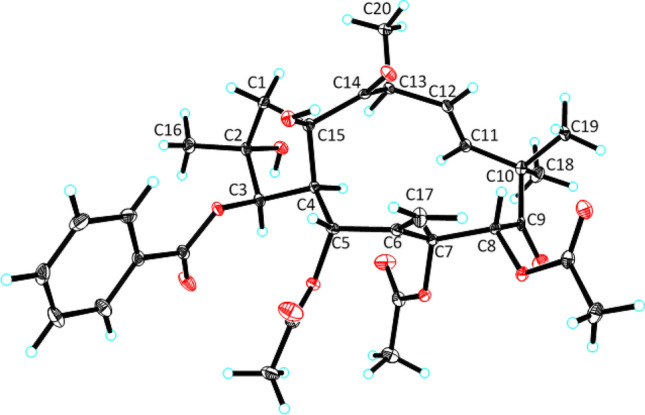
ORTEP drawing of compound **1**

Initially, we try to determine the relative configuration of **1** by a ROESY experiment (Fig. 2). We observed ROESY cross peaks of H-7/H-4, H-7/H-13, OH-2/H-3 and H-8/H_3_-19. However, the lack of correlation of the hydrogen atom at the five membered ring and the eleven membered ring led the establishment of the gross relative configuration difficult. Furthermore, the disappearance of H-5 signal made the assignment of its relative configuration impossible. Fortunately, after several attempts, crystals of **1** (CCDC: 2,043,474) were obtained, which allowed the assignment of the relative configuration of **1**. The absolute configuration of compound **1** was confirmed based on X-ray crystallography with Cu K*α* radiation resulted in the Flack parameter = 0.04 (4) (Fig. 3).

The molecular formula of euphopepluanone I (**2**) was determined to be C_36_H_44_O_12_ based on its positive HRESIMS ([M + Na]^+^, *m/z* 691.2716, calcd for C_36_H_44_O_12_Na, 691.2725). Comparison of the spectroscopic data of **2** with those of **1** suggested similar structure but with different esterification patterns. The former harbored an angeloxy group (*δ*_H_ 6.16, 2.01, 1.87) instead of the acetoxy group at C-7. HMBC correlations of the carbonyl carbons (*δ*_C_ 166.9) and oxymethine protons (*δ*_H_ 6.09) placed the angeloxy group at C-7. The relative configuration of **2** was the same as that of **1** by their similar ROESY cross-peaks.

The positive HRESIMS data of euphopepluanone J (**3**) showed an [M + Na]^+^ ion at *m/z* 693.2512 (calculated for 693.2518), corresponding to the molecular formular C_35_H_42_O_13_. The mass spectrum indicate compound **3** was 42 mass units more than compound **1**, suggesting that one of hydroxyl group in **1** was acetylated in compound **3**. On comparing its NMR data with those of **1** (Table 1), **3** displayed additional signals responsible for an acetoxy group (*δ*_H_ 2.19; *δ*_C_ 169.8, 22.3), and the absence of the OH-2 signal. Thus, the OH-2 in **1** was inferred as being replaced by an acetoxy group in **3**, which was supported by the HMBC correlations from H-2 to the carbonyl carbon. Therefore, the structure of **3** was delineated as shown.

**Fig. 4:**
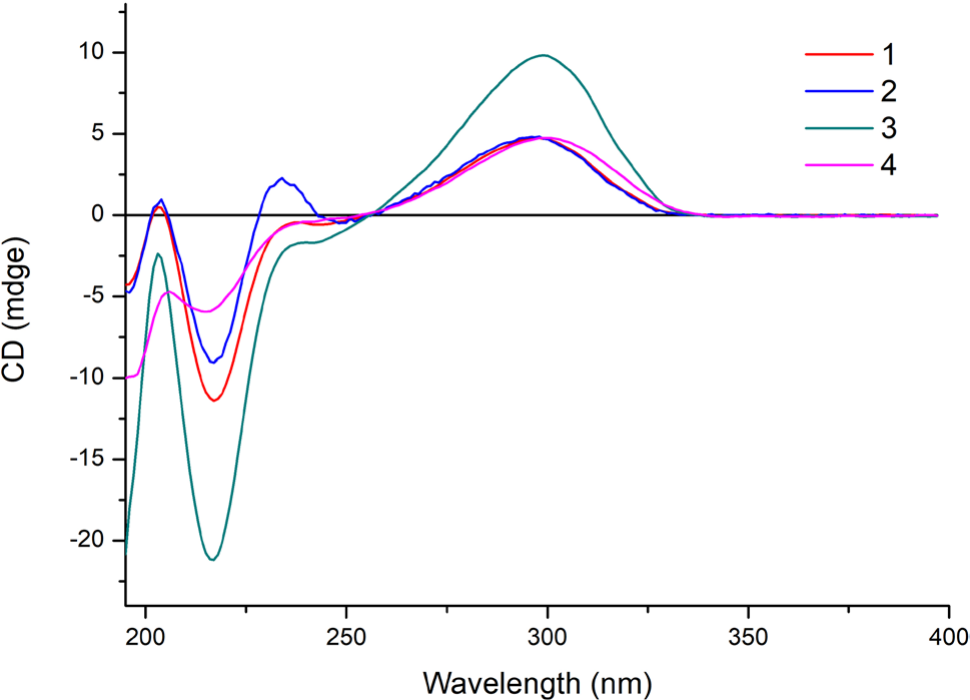
ECD spectra of compounds **1**−**4**

Euphopepluanone K (**4**) possessed a molecular formula of C_27_H_34_O_8_ as deduced from its positive HRESIMS ([M + Na]^+^, *m/z m/z* 509.2151, calcd for C_27_H_34_O_8_Na, 509.2146). The spectra data of **4** closely related to those of **5**, except for the absence of acetate signals at C-5, C-7, and C-8, suggesting the replacement of them with hydroxyl groups. Indeed, the resonances of C-4, C-6 and C-9 were up-shielded (Δ*δ*_C_ + 2.7, + 6.8, + 6.4 ppm), and C-5 and C-8 were down-shielded (Δ*δ*_C_ -0.7, -1.6) in **4**, further supporting the presence of hydroxy groups at C-5, C-7, and C-8, instead of acetoxy groups in compound **5**. The relative configuration of **4** was assigned as that of **1** by the nearly identical ROSEY data of these two compounds. Furthermore, the similar electronic circular dichroism (ECD) spectra of compounds **1**, **2**, **3** and** 4** (Fig. 4) indicated they share the same absolute configurations.

Eight known diterpenoids were characterized as hydroxyjatropha-6(17),11*E*-diene-9,14-dione (**5**) [17], 3,5,7,15-tetraacetoxy-9-nicotinoyloxy-14-oxojatropha-6(17),11-diene (**6**) [19], pepluanin D (**7**) [20], ingenol-20-angelate (**8**) [21], ingenol-3-angelate (**9**) [22], 5-*O*-benzoyl-20-deoxyingenol (**10**) [23], 5,8,9,15-tetraacetoxy-3-benzoyloxy-11,16-dihydroxypepluane (**11**) [24], and 5,8,9,11,15-pentaacetoxy-3-benzoyloxy-16-hydroxypepluane (**12**) [25] by comparing their spectroscopic data with those in the literatures.

### Bioactivity Evaluation

**Fig. 5:**
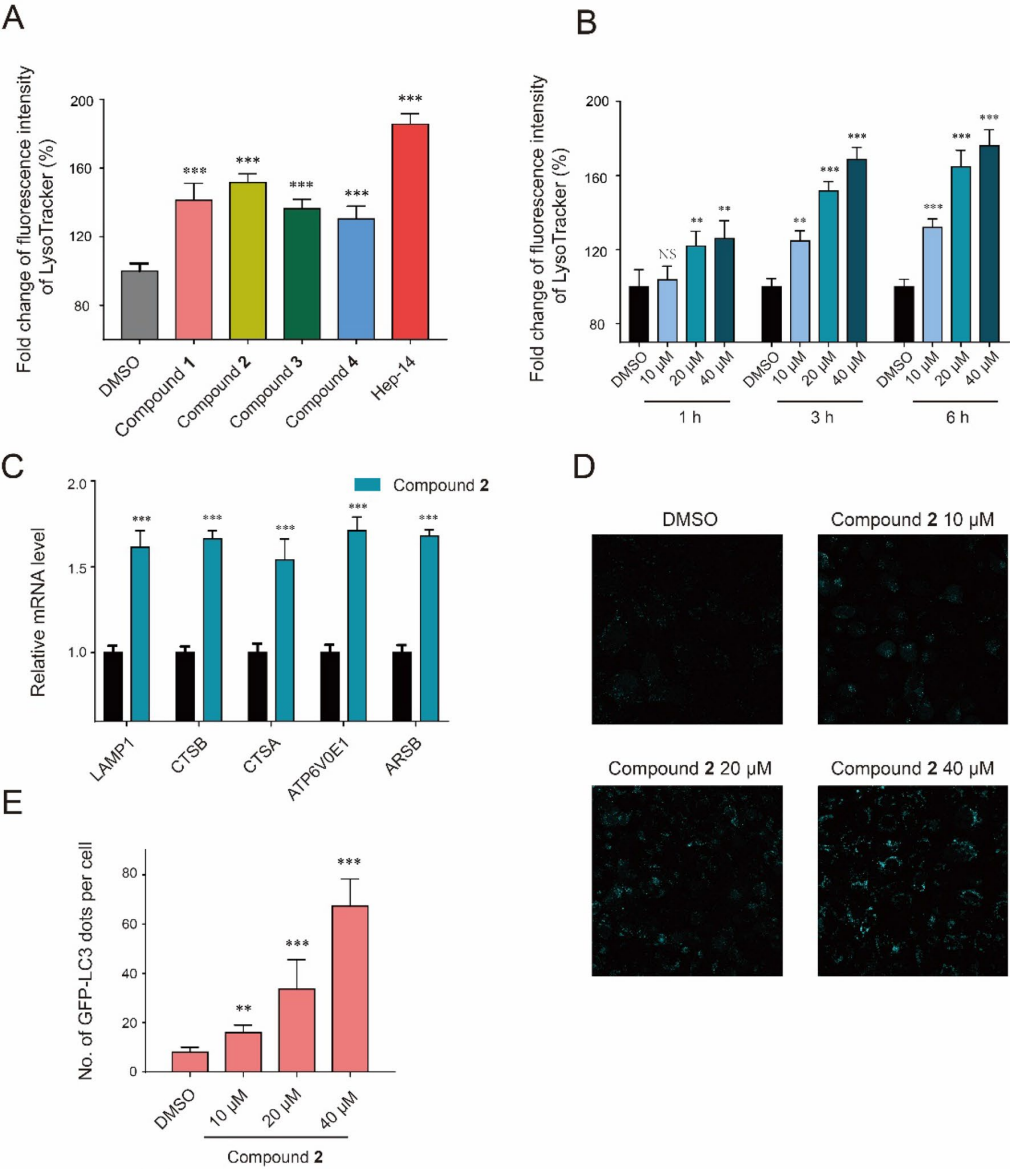
Compound **2** activates the lysosomal-autophagy pathway. **a** Compounds **1**−**4** induced lysosomal biogenesis. The number of lysosomes was stained with LysoTracker and mean fluorescence intensity (MFI) of LysoTracker was quantified. The bar graph showed the fold change of MFI of LysoTracker. Hep-14 served as positive control. **b** Compound **2** induced lysosomal biogenesis in a dose- and time-dependent manner. Cells were treated with compound **2** as indicated dose and time. The fold change of MFI of LysoTracker was analyzed. **c** Compound **2** induce the expression of lysosomal genes. HeLa cells were treated with compound **2** (20 μM, 3 h) and subjected to qRT-PCR analysis. The mRNA levels of lysosomal related genes were measured, and actin was used as an internal control. **d**, **e** Compound **2** increased the LC3 dots in a dose-dependent manner. The cells with CFP-LC3 were treated with compound **2** for 12 h with indicated concentration and the LC3 dots were imaged with confocal microscope. The representative images were shown in **d** and the quantification of LC3 dots were plotted in **e**. All experiments were carried out in triplicates and bar graph represents mean ± SD. *p* < 0.05 were considered statistically significant. **p < 0.01, ***p < 0.001

To assess the activity to enhance lysosomal biogenesis of the new compounds **1**–**4**, LysoTracker Red staining method was used. All the four new compounds increased the LysoTracker staining intensity. The cells were treated for 3 h with compounds **1** − **4** at 20 μM, and these compounds increased the LysoTracker staining intensity by 141.3%, 151.7%, 136.4% and 130.1%, respectively (Fig. 5a). Hep-14 was used as positive control [10]. It was further tested whether the lysosome biogenesis activities of these compounds are time- and concentration-dependent. As shown in Fig. 5b, HeLa cells were treated for 1, 3 and 6 h with 10, 20 and 40 μM of compound** 2** as indicated. Induction of lysosomes was observed in a time- and concentration-dependent manner, with the greatest increase at 40 μM when the cells were treated for 6 h. Many lysosomal genes were upregulated during lysosome biogenesis. To confirm that compound **2** induce lysosomal biogenesis, the expression levels of a set of lysosomal genes were checked, including lysosomal-associated membrane protein 1 (*LAMP1*), cathepsin B (*CTSB*), cathepsin A (*CTSA*), lysosomal sulfatase (*ARSB*), and ATPase H^+^ transporting V0 subunit E1 (*ATP6 V0E1*). As shown in Fig. 5c, all these genes were upregulated at mRNA levels 3 h after treatment with compound **2**. These data further demonstrated that compound **2** can induce lysosomal biogenesis. Then, we checked the level of LC3 dots, the marker for the activation of autophagy, induced by compound **2**. The number of LC3 dots increased with the treatment of compound **2** in a dose-dependent manner (Fig. 5d, e). These results indicate that compound **2** could activate the lysosomal-autophagy pathway.

## Experimental Section

### General Experimental Procedures

Optical rotations were measured with a Jasco P-1020 automatic polarimeter. CD spectra were obtained on the Applied Photophysics circular dichroism spectrometer (Applied Photophysics, Leatherhead, Surrey, UK). High-resolution MS data were measured on an Agilent 1290 UPLC/6540 Q-TOF mass spectrometer in positive mode. IR spectra were determined on a NICOLET iS107 Mid-infrared spectrometer. NMR spectra were measured on Bruker AVANCE III 500 MHz and AV 600 MHz NMR spectrometers with TMS as the internal standard. An Agilent 1260 series instrument equipped with a SunFire-C_18_ column (5 μm, 10 mm × 250 mm) and XSelect HSS T3(5 μm, 10 mm × 150 mm) were used for high-performance liquid chromatography (HPLC). Silica gel (100 − 200, 200 − 300, 300 − 400) mesh (Qingdao Marine Chemical, Inc), NH MB 100–40/75 Silica gel (FUJI SILYSIA CHEMICAL LTD), Lichroprep RP-18 (40 − 63 μm, Fuji), and Sephadex LH-20 (20 − 150 μm, Pharmacia) was used for CC.

### Plant Material

In August 2018, the seeds of *E. peplus* were collected from Kunming Botanical Garden, Yunnan Province, People’s Republic of China. A voucher specimen (No. kep-09–13) identified by Prof. Hu Shi-Jun (Southwest Forestry University) was deposited in the herbarium of the Kunming Institute of Botany, Chinese Academy of Sciences.

### Extraction and Isolation

The air-dried the seeds of *E. peplus* (24 kg) were powdered and extracted with methanol thrice at room temperature. The extract was suspended in water and extracted with petroleum ether, and ethyl acetate. The ethyl acetate extract (800 g) was subjected to a silica gel column using petroleum ether/ethyl acetate (100:0 to 0:100, v/v) as eluent to obtain 10 fractions, F1 − F10, in which diterpenes are mainly concentrated in F7 and F8.

Fraction F7 (58 g) was subjected to MCI gel developed with MeOH-H_2_O (40:60–100:0) to give 12 fractions. F7.7 (5.5 g) was submitted to silica gel column chromatography and eluted with ether/ethyl acetate gradient (20:1–1:1) to afford 7 fractions F7.7.1-F7.7.7. The fraction F7.7.3 (2.3 g) was isolated by amino-silica gel column (ether/ethyl acetate, 20:1–1:1) giving 8 fractions. Fraction F7.7.3.6 (99 mg) was purified by semi-preparative HPLC to yield compound **4** (2.4 mg). The fraction 7.7.4 (2.1 g) was separated by Sephadex LH-20 (MeOH/MeCl_3_ 50:50) to give four fractions, in which F7.7.4.2 (134 mg) was applied to preparative HPLC leading to compounds **3** (13.2 mg) and **5** (5.8 mg). F7.8 (6.3 g) was separated by silica gel column chromatography using ether/ethyl acetate gradient (20:1–1:1) to afford 8 fractions. Fraction F7.8.2 (1.5 g) and F7.8.3 (0.6 g) were chromatographed on Sephadex LH-20 (MeOH/MeCl_3_ 50:50) to afford five and seven fractions, respectively. F7.8.2.5 (29 mg) and F7.8.3.2 (42 mg) were each submitted to semi-preparative HPLC to give compounds **10** (4.8 mg) and **2** (6.7 mg), respectively.

Fraction F8 (66 g) was subjected to MCI gel with MeOH-H_2_O (40:60–100:0) to give 13 fractions, in which compound **1** (378.3 mg) was obtained from fraction 7 as colorless crystals. Fraction F8.7 (12 g) was submitted to silica gel column with ether/ethyl acetate gradient (20:1- 1:1) leading to 6 fractions, in which fraction F8.7.1 (218 mg) was further purified by silica gel column with gradient of ether/ethyl acetate (10:1–1:1) to give five fractions F8.7.1.1-F8.7.1.5, and then the fraction F8.7.1.5 (33.7 mg) was subjected to preparative HPLC to yield compound **7** (22.3 mg). Fraction F8.7.3 (3.1 g) was chromatographed on Sephadex LH-20 (MeOH/MeCl_3_ 50:50) giving three fractions. The resulted F8.7.3.1 (400 mg) was purified by silica gel column and eluted with ether/ethyl acetate (10:1–5:1) to afford six fractions, and **11** (42.7 mg) was obtained from fraction F8.7.3.1.2 by preparative HPLC. Fraction F8.7.3.2 (2.3 g) was chromatographed on silica gel column with ether/ethyl acetate (1:5) leading to seven fractions. The obtained fraction F8.7.3.2.1 (141 mg) was purified by preparative HPLC to yield compound **8** (51.9 mg). The fraction F8.8 (7.9 g) was submitted to Sephadex LH-20 (MeOH/MeCl_3_ 50:50) to give six fractions, in which fraction F8.8.1 (6.1 g) was separated by silica gel column with gradient of ether/ethyl acetate (10:1–5:1) to afford 10 fractions. The fractions F8.8.1.4 (43 mg) and F8.8.1.6 (50 mg) were each purified by preparative HPLC producing compounds **12** (24.4 mg) and **6** (9.1 mg), respectively. Fraction F8.8.3 (695 mg) was chromatographed on with gradient of ether/ethyl acetate (10:1–5:1) to give eight fractions, in which F8.8.3.6 (51 mg) was further separated by preparative HPLC leading to compound **9** (16.8 mg).

Euphopepluanone F (**1**): a colorless massive crystal (MeOH/H_2_O, 20/1); mp 216–220 °C; [*α*]_D_^25^ + 64.9 (*c* 0.11, MeOH); UV (MeOH) *λ*_max_ (log *ε*) 195 (4.52), 229 (3.96) nm; IR (KBr) *v*_max_ 3481, 2977, 1743, 1723, 1650, 1454, 1374, 1277, 1224 cm ^−1^; ^1^H and ^13^C NMR data, see Table 1; ( +)-HRESIMS *m/z* [M + Na]^+^ 651.2426 (calcd for C_33_H_40_O_12_Na, 651.2412).

Euphopepluanone I (**2**): a white amorphous powder; [*α*]_D_^25^ + 41.2 (*c* 0.13, MeOH); UV (MeOH) *λ*_max_ (log *ε*) 195 (4.48), 226 (4.05) nm; IR (KBr) *v*_max_ 3436, 2975, 2934, 1748, 1726, 1644, 1453, 1374, 1273 cm ^−1^; ^1^H and ^13^C NMR data, see Table 1; ( +)-HRESIMS *m/z* [M + Na]^+^ 691.2716 (calcd for C_36_H_44_O_12_Na, 691.2725).

Euphopepluanone J (**3**): a white amorphous powder; [*α*]_D_^25^ + 16.3 (*c* 0.08, MeOH); UV (MeOH) *λ*_max_ (log *ε*) 195 (4.54), 229 (3.91) nm; IR (KBr) *v*_max_ 3447, 2981, 1747, 1728, 1633, 1453, 1372, 1239 cm ^−1^; ^1^H and ^13^C NMR data, see Table 1; ( +)-HRESIMS *m/z* [M + Na]^+^ 693.2512 (calcd for C_35_H_42_O_13_Na, 693.2518).

Euphopepluanone K (**4**): a white amorphous powder; [*α*]_D_^25^ + 59.3 (*c* 0.11, MeOH); UV (MeOH) *λ*_max_ (log *ε*) 195 (4.45), 226 (3.92) nm; IR (KBr) *v*_max_ 3436, 2970, 2933, 1710, 1631, 1452, 1380, 1278 cm ^−1^; ^1^H and ^13^C NMR data, see Table 1; ( +)-HRESIMS *m/z* [M + Na]^+^ 509.2151 (calcd for C_27_H_34_O_8_Na, 509.2146).

### X-ray Crystallographic Analyses

Crystallographic Data for Compound **1.** C_33_H_40_O_12_, *M* = 628.65, *a* = 10.0011(3) Å, *b* = 17.0114(4) Å, *c* = 10.2133(3) Å, *α* = 90°, *β* = 114.3100(10)°, *γ* = 90°, *V* = 1583.54(8) Å^3^, *T* = 100(2) K, space group *P*21, *Z* = 2, *μ*(CuK*α*) = 0.839 mm^−1^, 17,488 reflections measured, 5524 independent reflections (*R*_*int*_ = 0.0390). The final *R*_*1*_ values were 0.0330 (*I* > 2*σ*(*I*)). The final *wR*(*F*^2^) values were 0.0852 (*I* > 2*σ*(*I*)). The final *R*_*1*_ values were 0.0330 (all data). The final *wR*(*F*^2^) values were 0.0853 (all data). The goodness of fit on *F*^2^ was 1.065. Flack parameter = 0.04(4). These data can be obtained free of charge from The Cambride Crystallographic Data Centre via http://www.ccdc.cam.ac.uk/data_request/cif.

### Cell Culture

The activity to enhance lysosomal biogenesis of compounds **1**–**4** was evaluated using HeLa cell line, which was cultured at 37 °C with 5% CO_2_ in Dulbecco’s modified Eagle’s medium supplemented with 10% fetal bovine serum (HyClone), 100,000 U/mL penicillin and 100 mg/mL streptomycin. HeLa cell was purchased from ATCC.

### Screening for Compounds That Induce Lysosomal Biogenesis

Briefly, HeLa cells with 85% cell density in 96-well plates were treated with individual compounds at 20 μM in triplicate. Three hours later, cells were grown in fresh medium containing LysoTracker Red DND-99 (0.2 μM) for 30 min. Then, medium was changed to LysoTracker-free medium and images were taken with ArrayScan Infinity (Cellomics, ArrayScan VTI HCS). Positive compounds were subjected to validation by treating HeLa cells with different concentrations (10, 20 and 40 μM) and at 1, 3 and 6 h in triplicate and staining with LysoTracker Red DND-99.

### Confocal Microscopy

CFP-LC3 expressing HeLa cells were treated with indicated compounds and images were collected by confocal microscopy. For live-cell imaging, cells grown on glass-bottom dishes were observed directly. All samples were examined with an inverted Olympus FV1000 confocal microscope. Images were analyzed with FV10-ASW 4.0a Viewer.

### Quantitative Real-Time PCR with Reverse Transcription (qRT-PCR)

Total RNA was isolated from HeLa cells by using TRIzol Reagent (Invitrogen) according to the manufacturer’s recommendation. A reverse-transcription kit (Promega) was used to reverse transcribe RNA (1 µg) in a 20 *µ*L reaction mixture. A real-time PCR system (7900HT Fast; Applied Biosystems) was used to quantify gene expression in triplicate. Amplification of the sequence of interest was normalized with the reference endogenous gene actin.

### Statistics and Reproducibility

Data analyses were carried out using Prism 5, and Student’s *t* tests were employed for statistical analyses with a level of significance of *p* < 0.05.

## Supplementary Information

Below is the link to the electronic supplementary material.

Supplementary file1 (DOCX 76991 kb)
