# Supplementary material for: Jatrophane Diterpenoids from the Seeds of Euphorbia peplus with Potential Bioactivities in Lysosomal-Autophagy Pathway
Source: Nat Prod Bioprospect. 2021 Mar 14;11(3):357–64. doi: 10.1007/s13659-021-00301-4 (PMC8140953; doi:10.1007/s13659-021-00301-4)
Supplement: Supplementary file 1 — Supplementary file1 (DOCX 76991 kb) [file 13659_2021_301_MOESM1_ESM.docx]

Supporting Information

**Jatrophane Diterpenoids from the Seeds of *Euphorbia peplus* with Potential** **Bioactivities in Lysosomal-Autophagy Pathway**

Yan-Ni Chen^a,1^, Xiao Ding^a,1^, Dong-Mei Li^a,c^, Qing-Yun Lu^a,b^, Shuai Liu^a,b^, Ying-Yao Li^a,c^, Ying-Tong Di^a^, Xin Fang^a^*, and Xiao-Jiang Hao^a^*

*^a^ State Key Laboratory of Phytochemistry and Plant Resources in West China, Kunming Institute of Botany, Chinese Academy of Sciences, Kunming, P.R. China;*

*^b^University of Chinese Academy of Sciences, Beijing, 100049, PR China*

*^c^Yunnan University, Kunming, P.R. China*

***Corresponding authors. E-mail address: xinfang@mail.kib.ac.cn (X. Fang), or haoxj@mail.kib.ac.cn (X.-J. Hao).

^1^ These authors contributed equally to this work.

Contents of Supporting Information

Contents

[**Figure 1.**^1^H NMR spectrum of compound **1** recorded inCDCl_3_ at 500 MHz 4](#_Toc45897998)

[**Figure 2.**^13^C NMR spectrum of compound **1** recorded inCDCl_3_ at 500 MHz 4](#_Toc45897999)

[**Figure 3.** HSQC spectrum of compound **1** recorded in CDCl_3_ 5](#_Toc45898000)

[**Figure 4.** ^1^H-^1^H COSY spectrum of compound **1** recorded in CDCl_3_ 5](#_Toc45898001)

[**Figure 5.** HMBC spectrum of compound **1** recorded in CDCl_3_ 6](#_Toc45898002)

[**Figure 6.** ROESY spectrum of compound **1** recorded in CDCl_3_ 6](#_Toc45898003)

[**Figure 7.** HRESIMS spectrum of compound **1** recorded in MeOH 7](#_Toc45898004)

[**Figure 8.** IR spectrum of compound **1** 7](#_Toc45898005)

[**Figure 9.** UV spectrum of compound **1** 8](#_Toc45898006)

[**Figure 10.**^1^H NMR spectrum of compound **2** recorded inCDCl_3_ at 500 MHz 8](#_Toc45898008)

[**Figure 11.**^13^C NMR spectrum of compound **2** recorded inCDCl_3_ at 500 MHz 9](#_Toc45898009)

[**Figure 12.** HSQC spectrum of compound **2** recorded in CDCl_3_ 9](#_Toc45898010)

[**Figure 13.** ^1^H-^1^H COSY spectrum of compound **2** recorded in CDCl_3_ 10](#_Toc45898011)

[**Figure 14.** HMBC spectrum of compound **2** recorded in CDCl_3_ 10](#_Toc45898012)

[**Figure 15.** ROESY spectrum of compound **2** recorded in CDCl_3_ 11](#_Toc45898013)

[**Figure 16.** HRESIMS spectrum of compound **2** recorded in MeOH 11](#_Toc45898014)

[**Figure 17.** IR spectrum of compound **2** 12](#_Toc45898015)

[**Figure 18.** UV spectrum of compound **2** 12](#_Toc45898016)

[**Figure 19.**^1^H NMR spectrum of compound **3** recorded inCDCl_3_ at 500 MHz 13](#_Toc45898017)

[**Figure 20.**^13^C NMR spectrum of compound **3** recorded inCDCl_3_ at 500 MHz 13](#_Toc45898018)

[**Figure 21.** HSQC spectrum of compound **3** recorded in CDCl_3_ 14](#_Toc45898019)

[**Figure 22.** ^1^H-^1^H COSY spectrum of compound **3** recorded in CDCl_3_ 14](#_Toc45898020)

[**Figure 23.** HMBC spectrum of compound **3** recorded in CDCl_3_ 15](#_Toc45898021)

[**Figure 24.** ROESY spectrum of compound **3** recorded in CDCl_3_ 15](#_Toc45898022)

[**Figure 25.** HRESIMS spectrum of compound **3** recorded in MeOH 16](#_Toc45898023)

[**Figure 26.** IR spectrum of compound **3** 16](#_Toc45898024)

[**Figure 27.** UV spectrum of compound **3** 17](#_Toc45898025)

[**Figure 28.**^1^H NMR spectrum of compound **4** recorded inCDCl_3_ at 500 MHz 17](#_Toc45898026)

[**Figure 29.**^13^C NMR spectrum of compound **4** recorded inCDCl_3_ at 500 MHz 18](#_Toc45898027)

[**Figure 30.** HSQC spectrum of compound **4** recorded in CDCl_3_ 18](#_Toc45898028)

[**Figure 31.** ^1^H-^1^H COSY spectrum of compound **4** recorded in CDCl_3_ 19](#_Toc45898029)

[**Figure 32.** HMBC spectrum of compound **4** recorded in CDCl_3_ 19](#_Toc45898030)

[**Figure 33.** ROESY spectrum of compound **4** recorded in CDCl_3_ 20](#_Toc45898031)

[**Figure 34.** HRESIMS spectrum of compound **4** recorded in MeOH 20](#_Toc45898032)

[**Figure 35.** IR spectrum of compound **4** 21](#_Toc45898033)

[**Figure 36.** UV spectrum of compound **4** 21](#_Toc45898034)

[**Table 1.** Crystal data and structure refinement for compound **1** 22](#_Toc45898057)


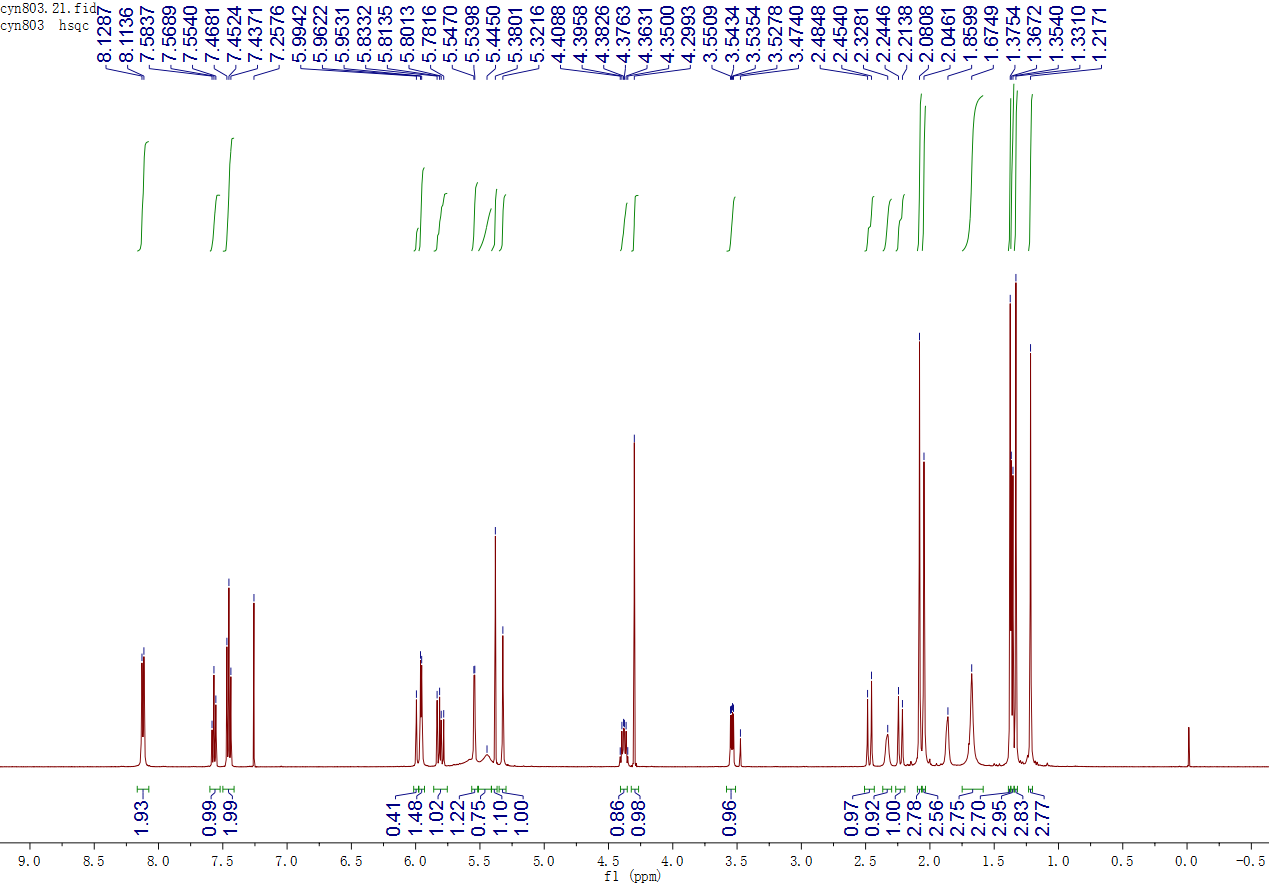


**Figure 1.**^1^H NMR spectrum of compound **1** recorded inCDCl_3_ at 500 MHz


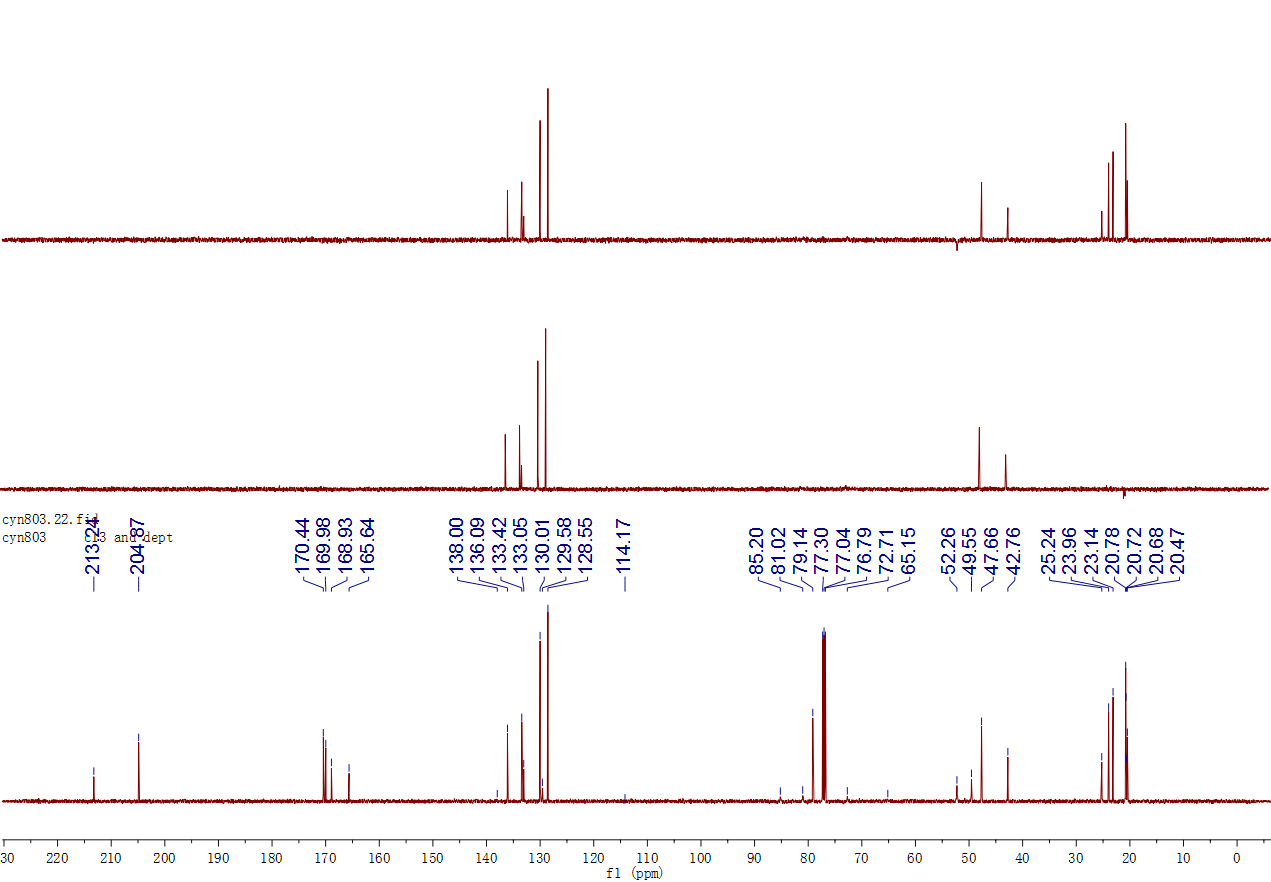


**Figure 2.**^13^C NMR spectrum of compound **1** recorded inCDCl_3_ at 500 MHz


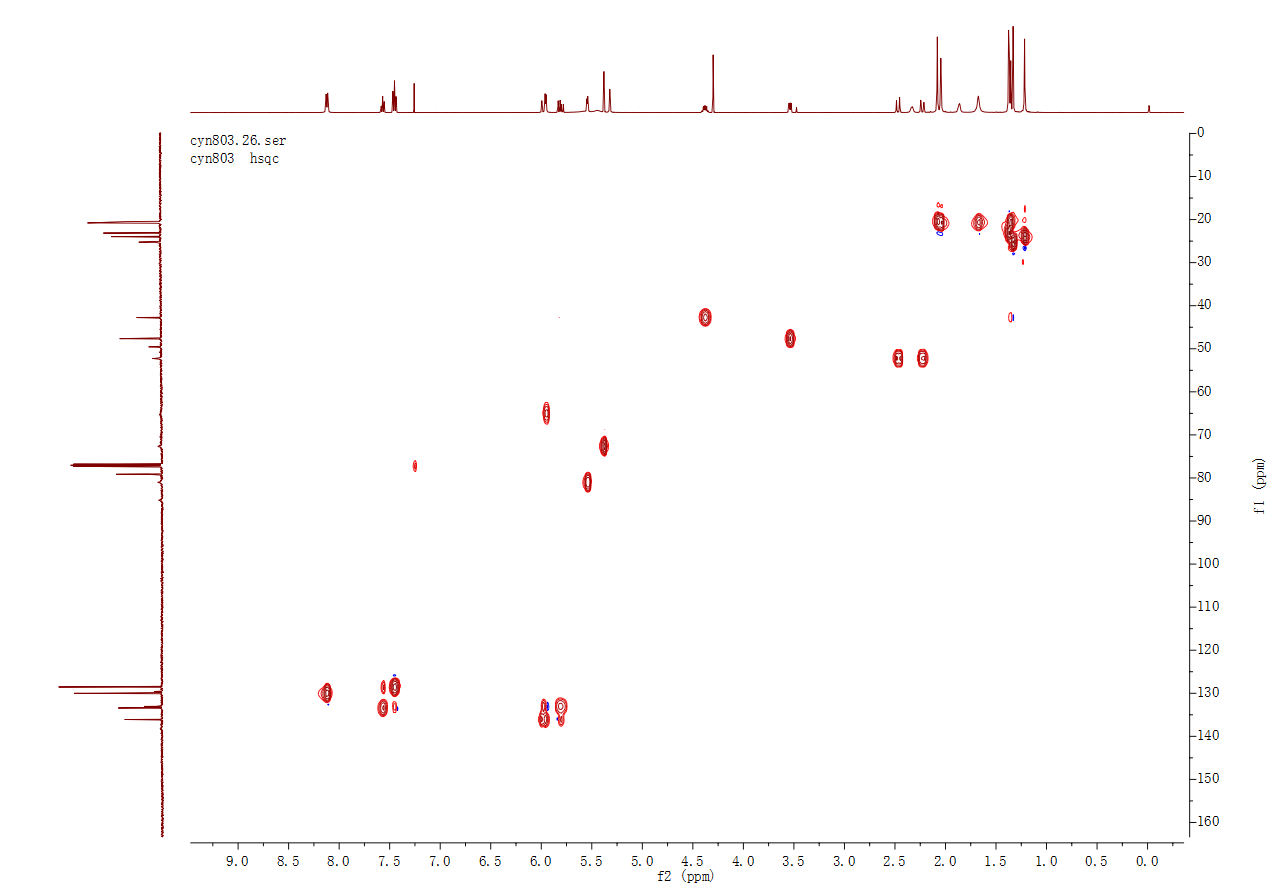


**Figure 3.** HSQC spectrum of compound **1** recorded in CDCl_3_


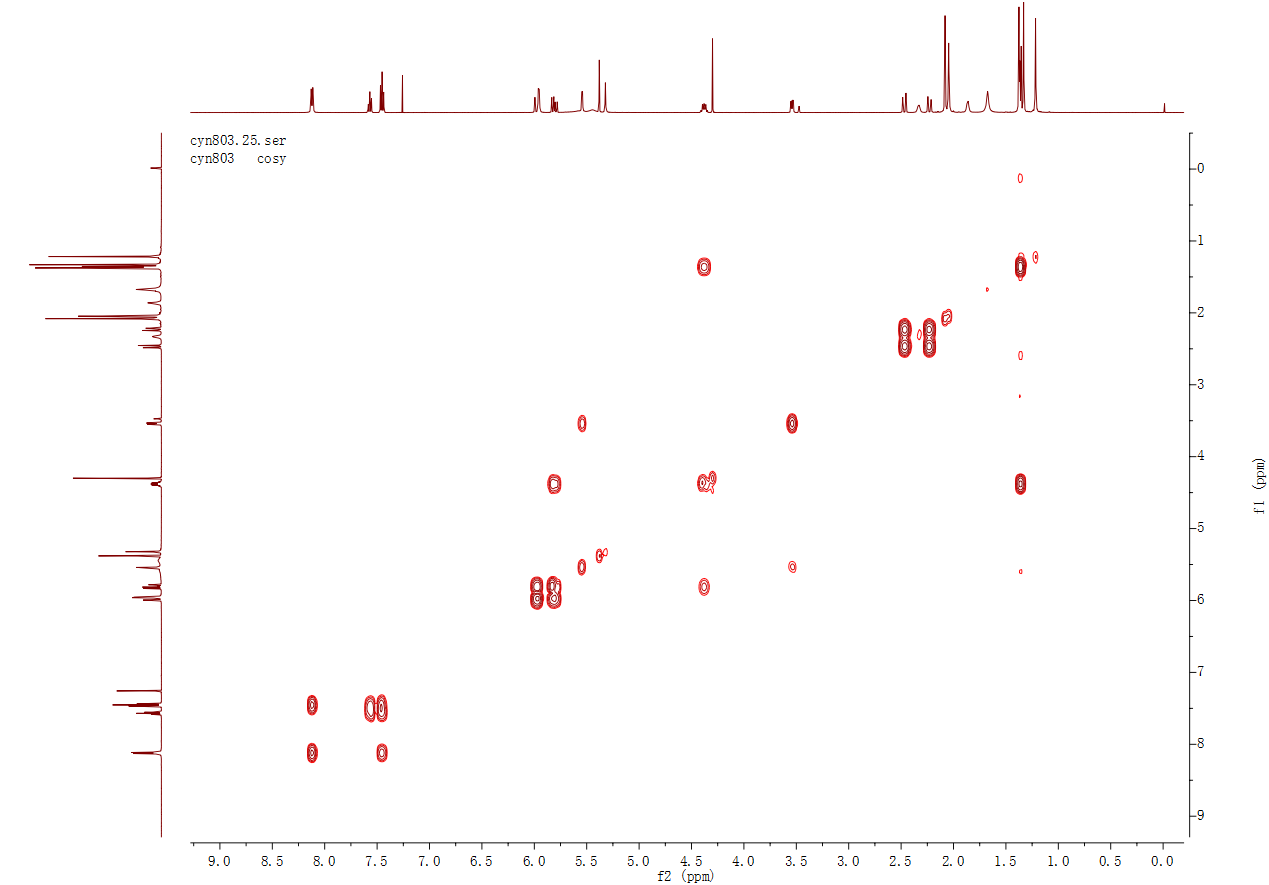


**Figure 4.** ^1^H-^1^H COSY spectrum of compound **1** recorded in CDCl_3_


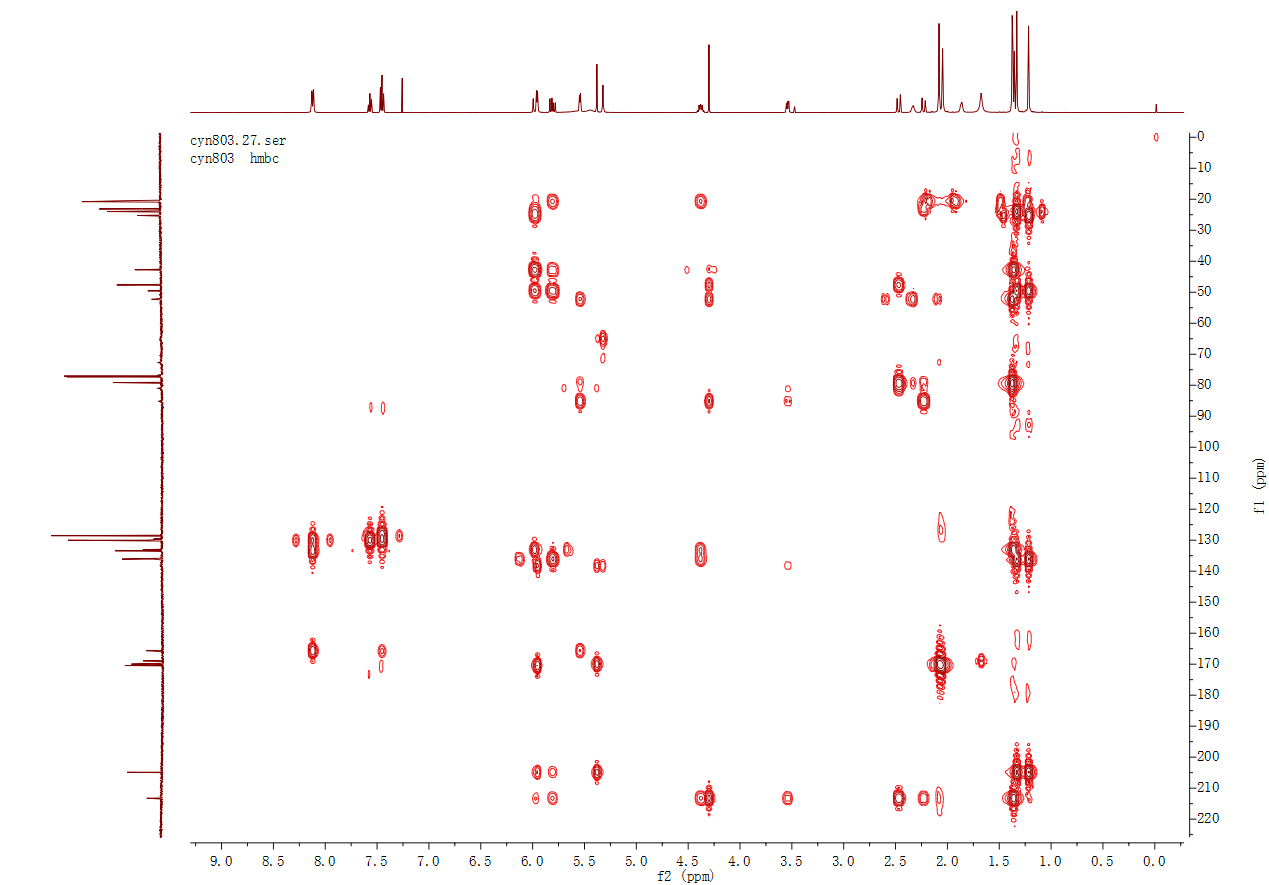


**Figure 5.** HMBC spectrum of compound **1** recorded in CDCl_3_


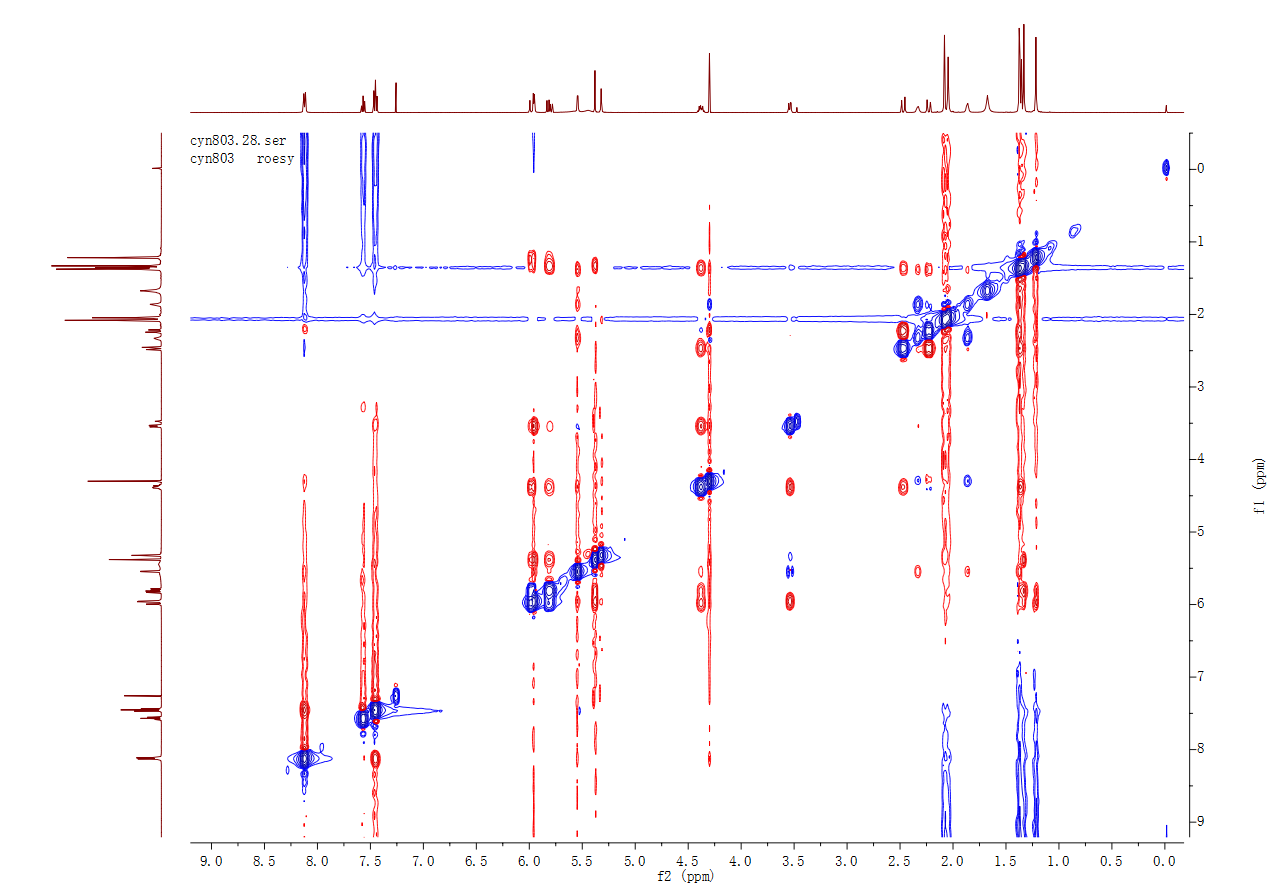


**Figure 6.** ROESY spectrum of compound **1** recorded in CDCl_3_


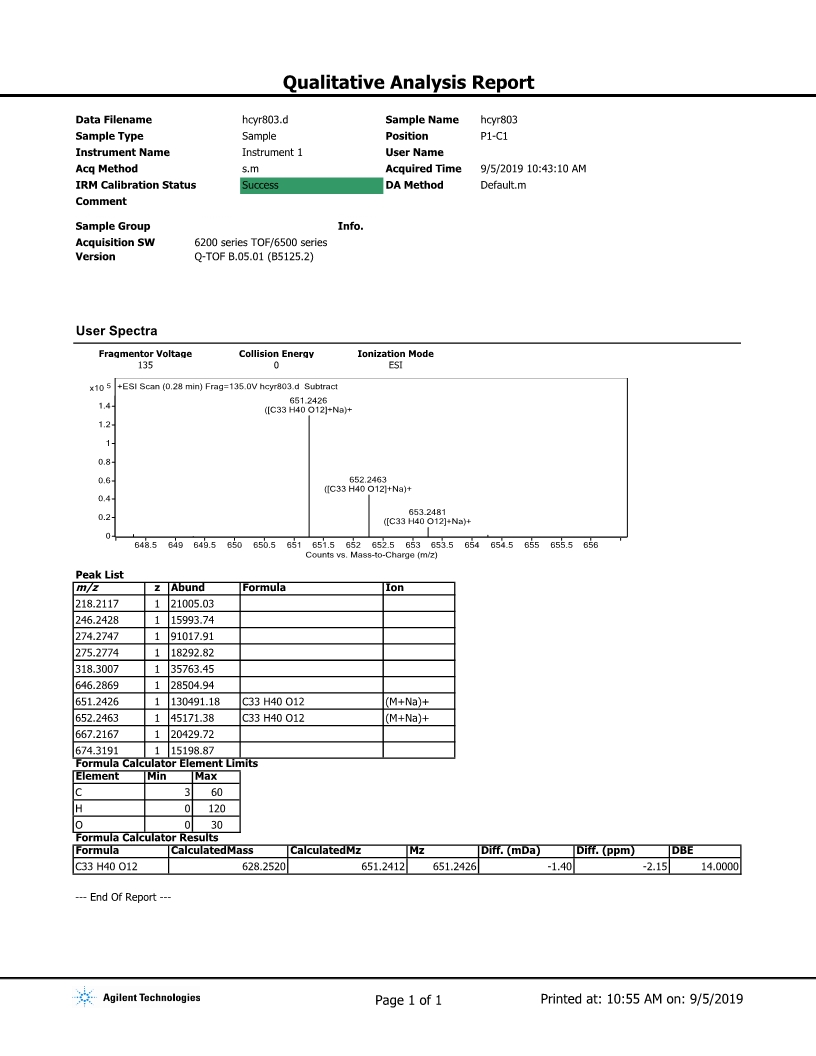


**Figure 7.** HRESIMS spectrum of compound **1** recorded in MeOH

**Figure 8.** IR spectrum of compound **1**


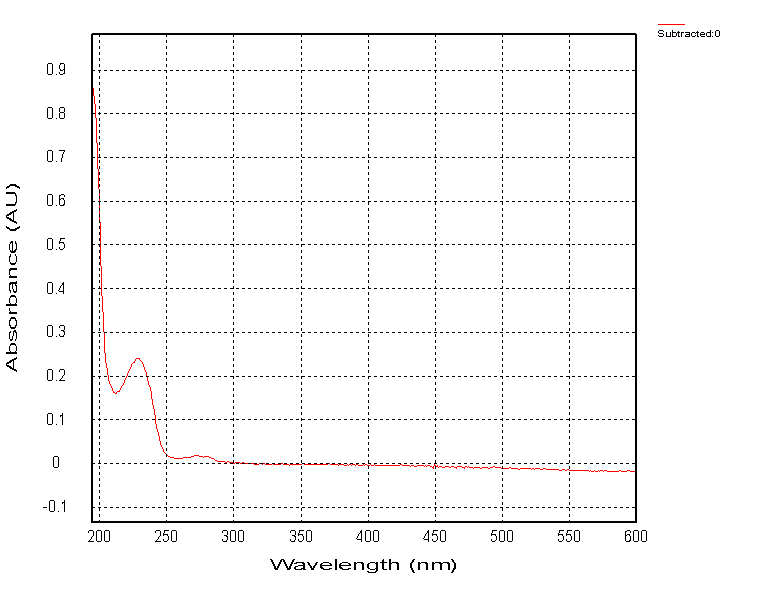


**Figure 9.** UV spectrum of compound **1**


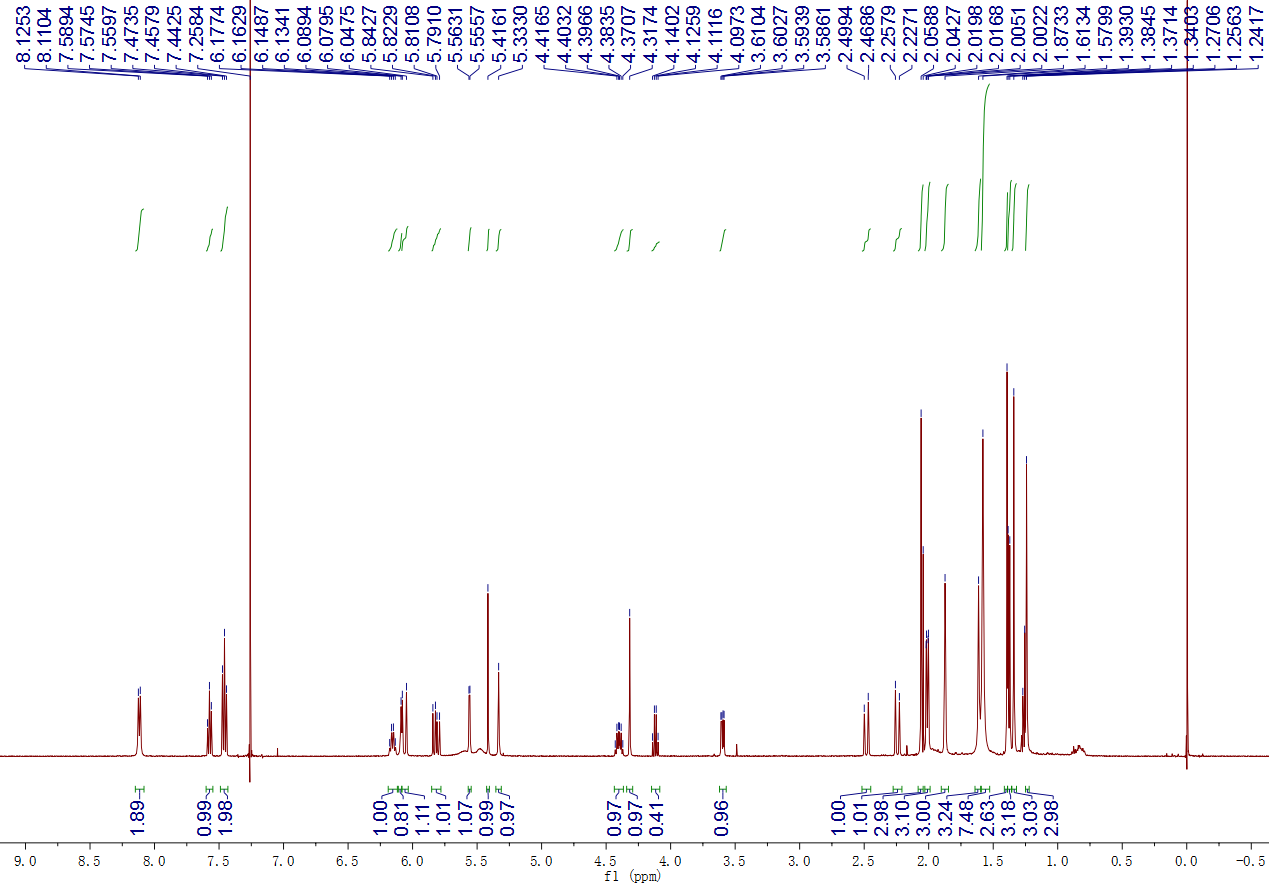


**Figure 10.**^1^H NMR spectrum of compound **2** recorded in CDCl_3_ at 500 MHz


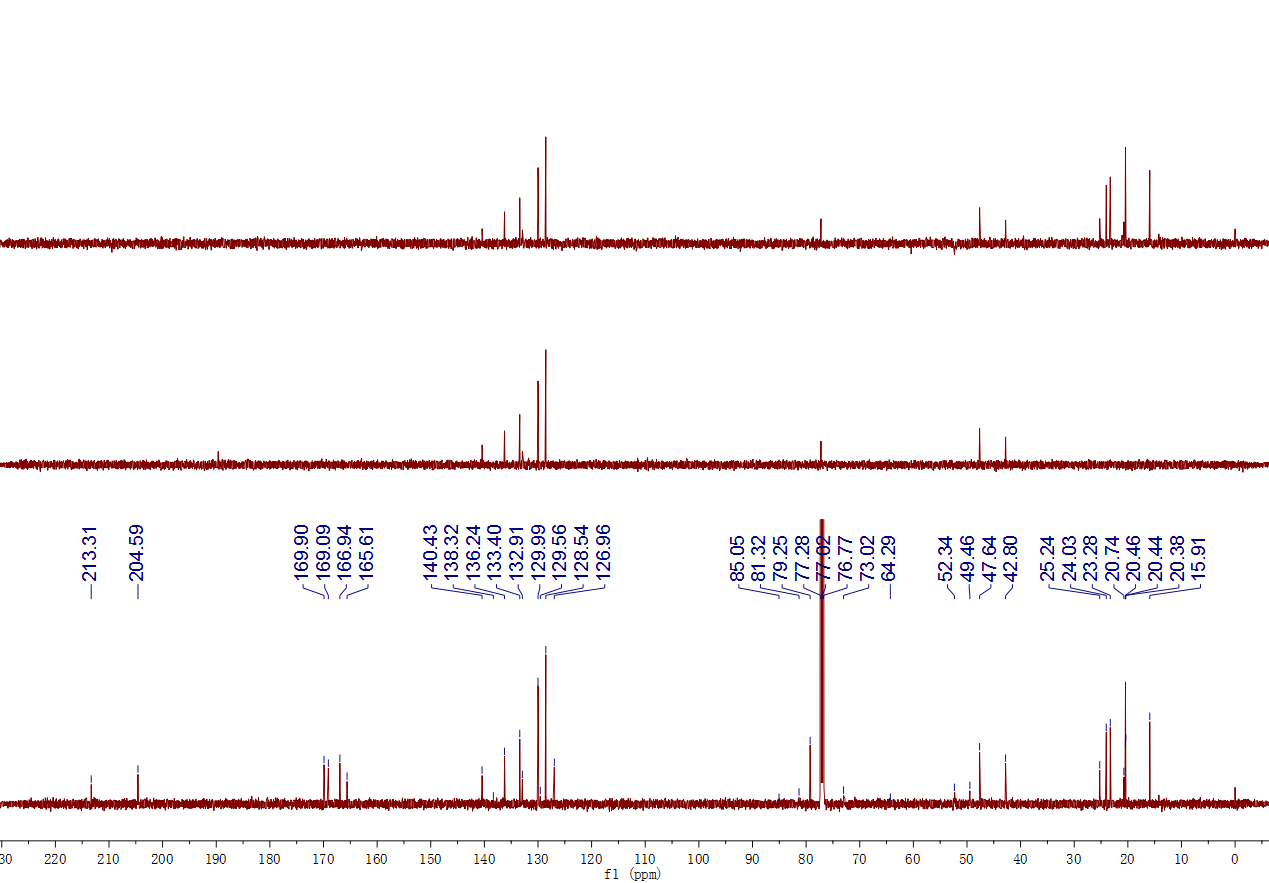


**Figure 11.**^13^C NMR spectrum of compound **2** recorded in CDCl_3_ at 500 MHz


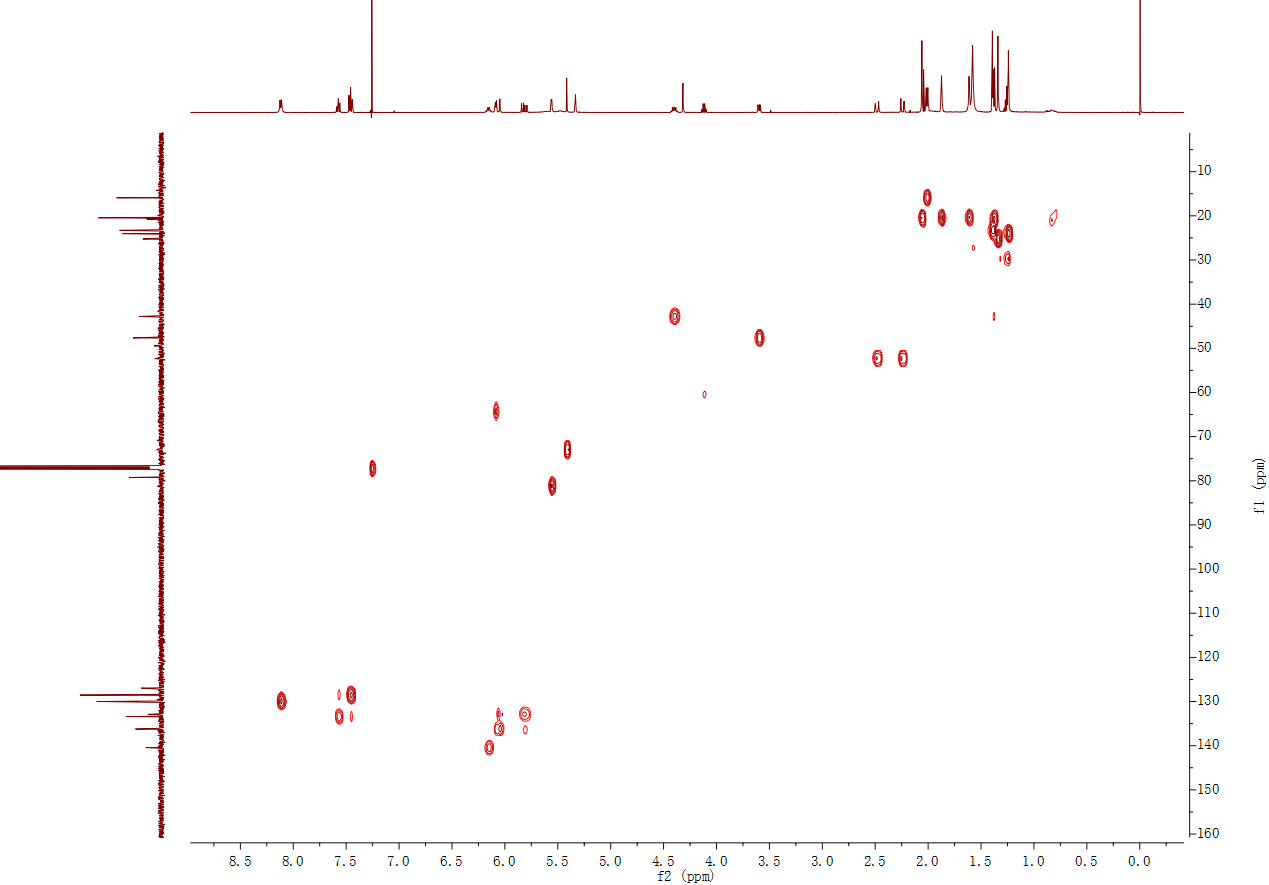


**Figure 12.** HSQC spectrum of compound **2** recorded in CDCl_3_


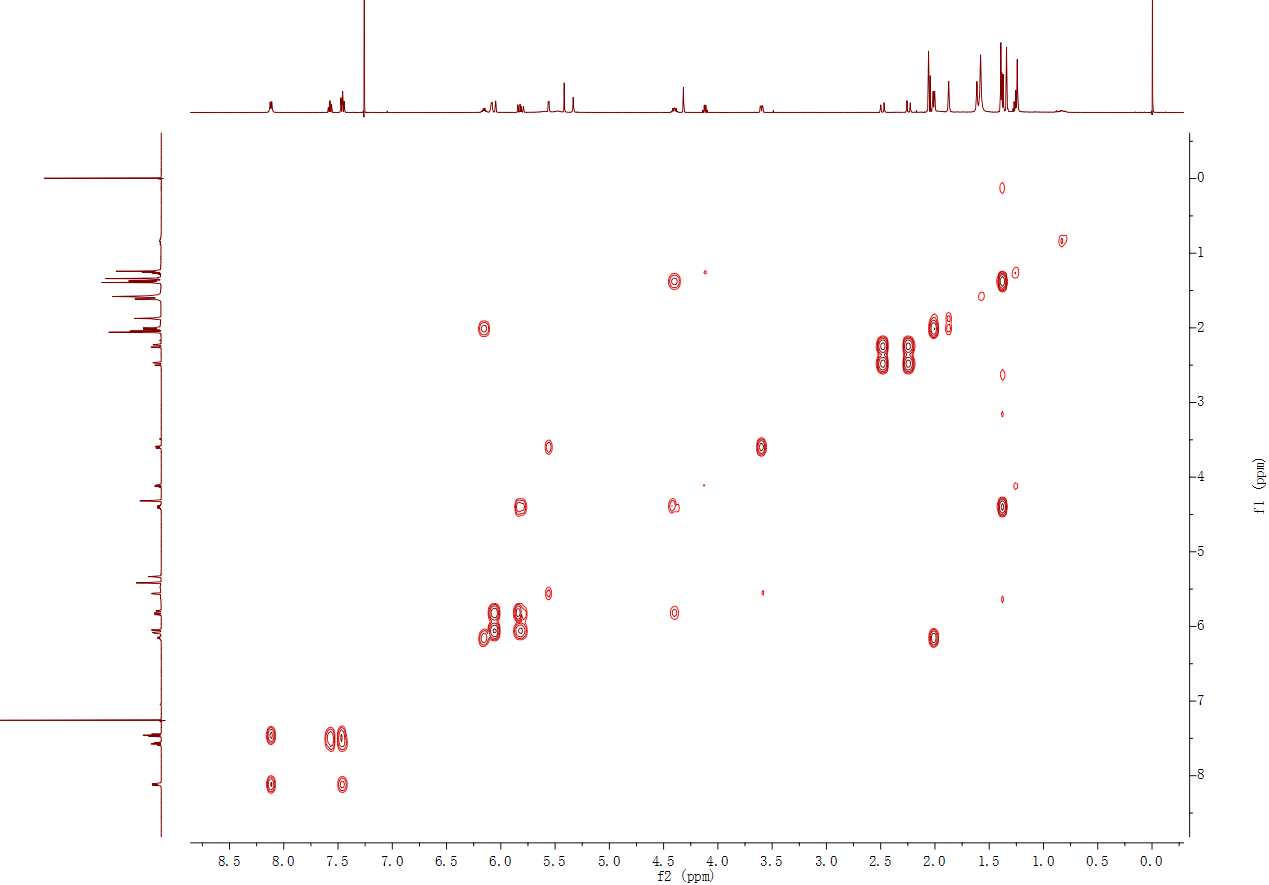


**Figure 13.** ^1^H-^1^H COSY spectrum of compound **2** recorded in CDCl_3_


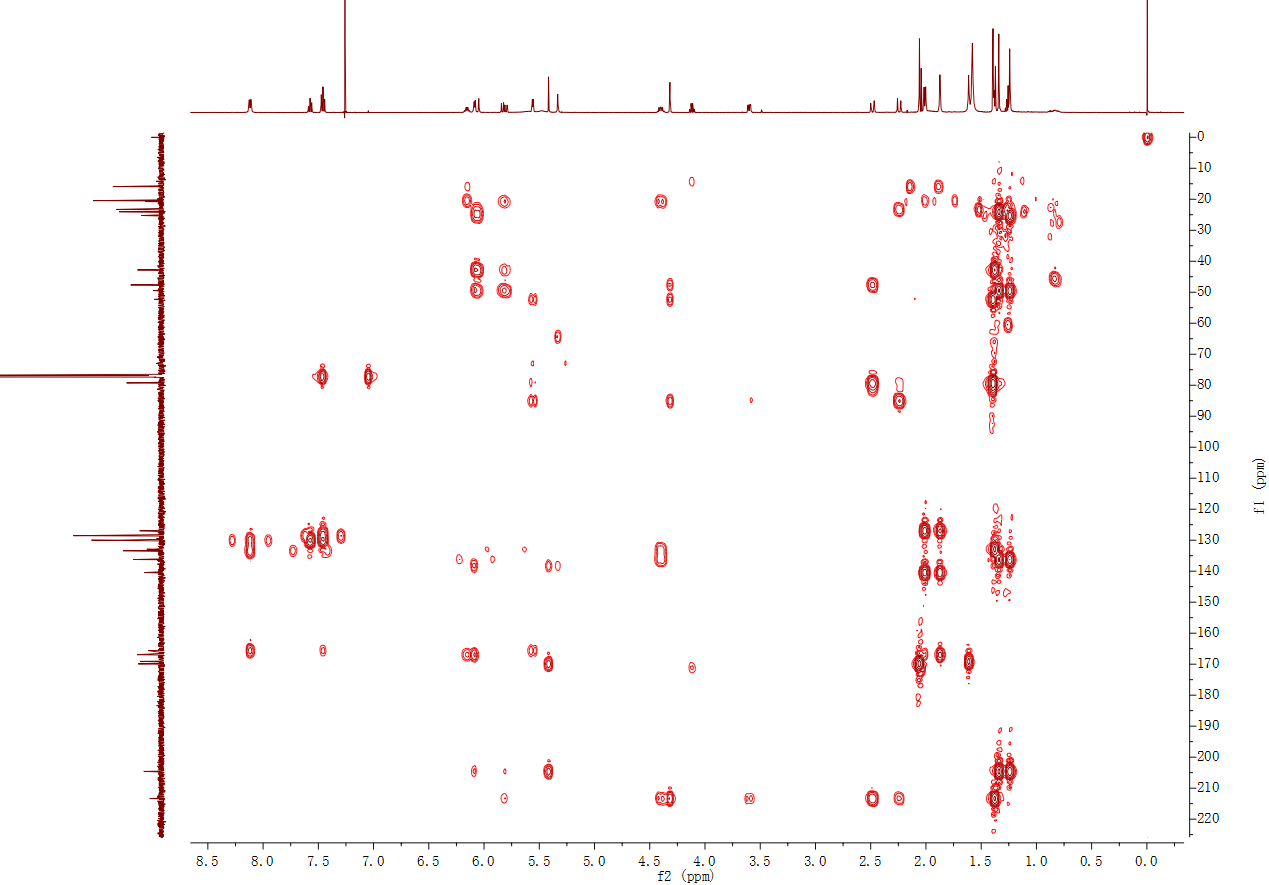


**Figure 14.** HMBC spectrum of compound **2** recorded in CDCl_3_


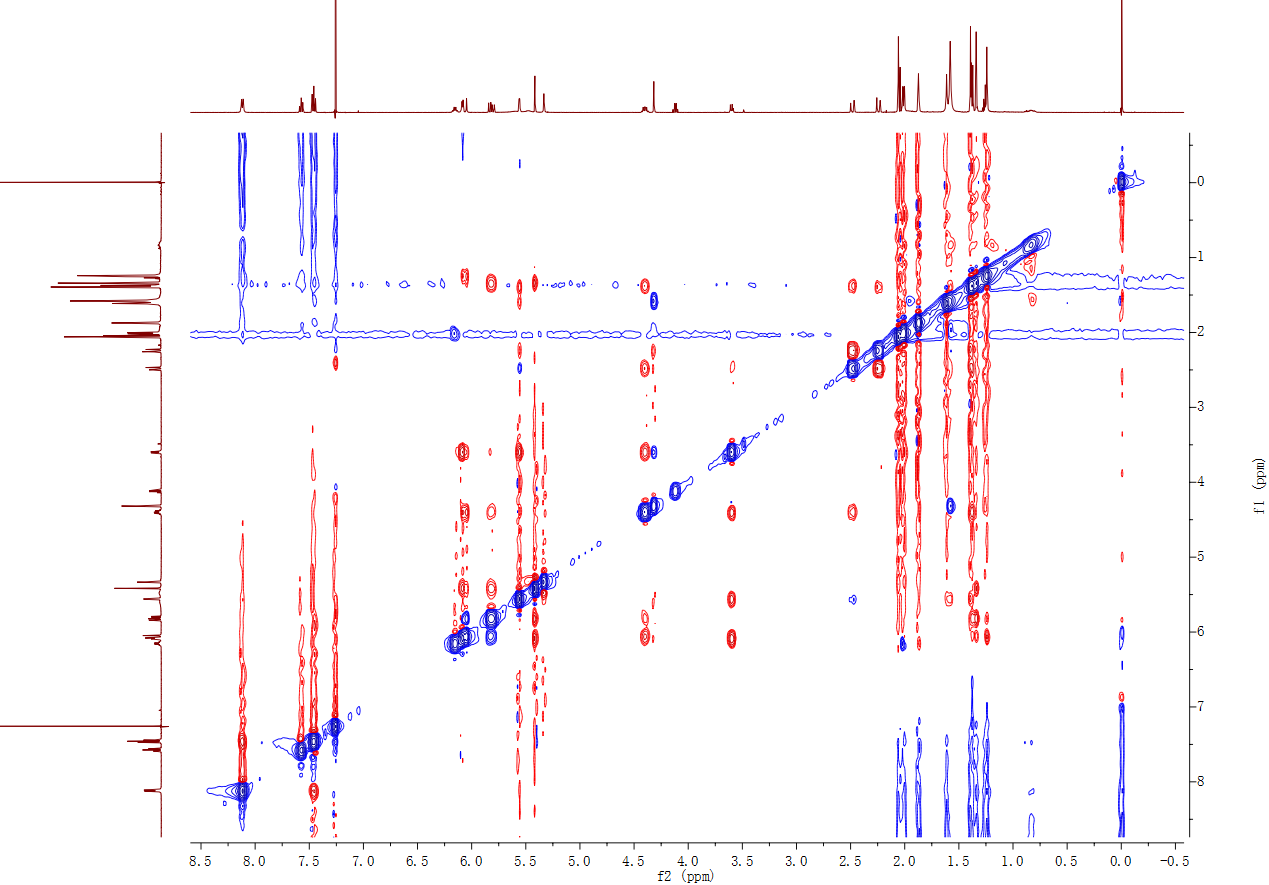


**Figure 15.** ROESY spectrum of compound **2** recorded in CDCl_3_


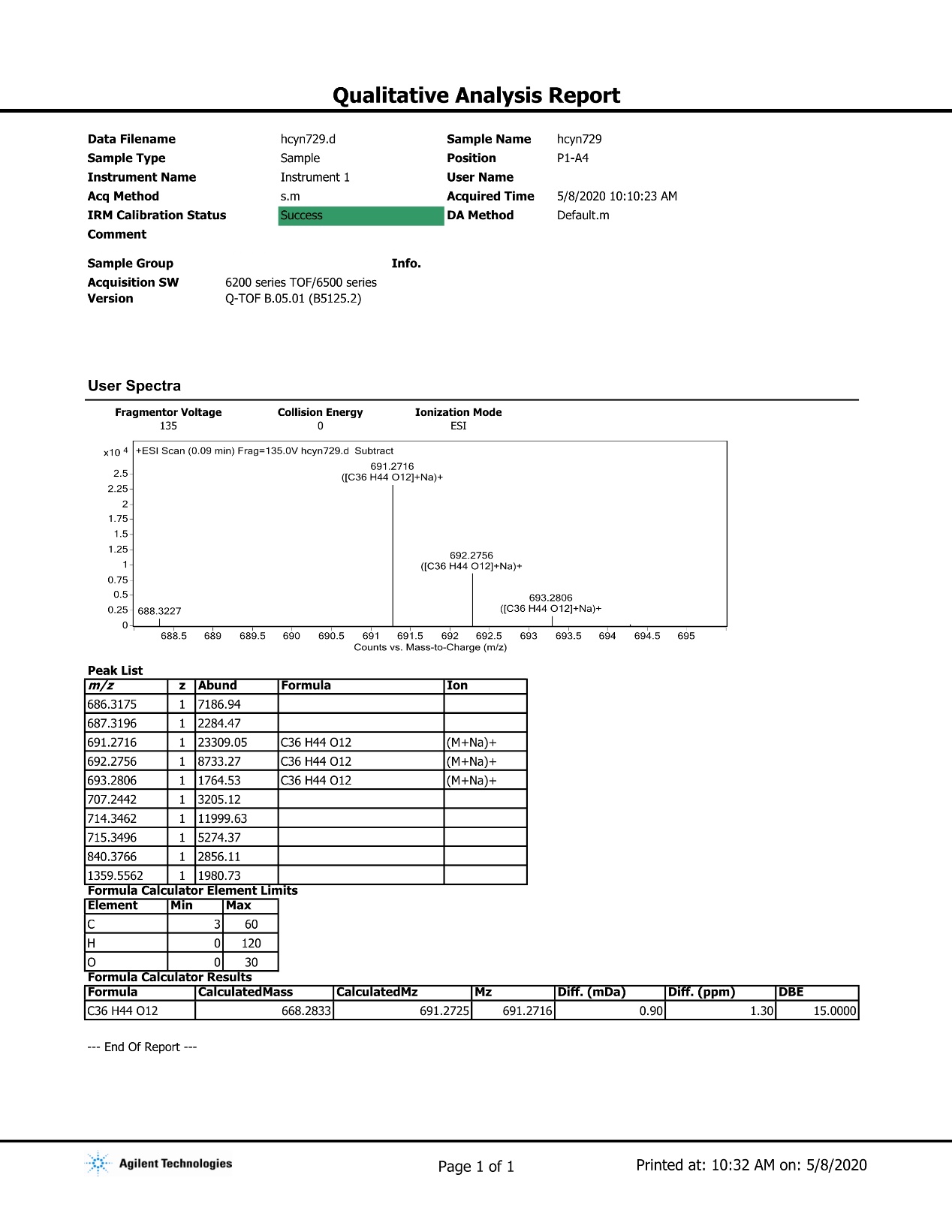


**Figure 16.** HRESIMS spectrum of compound **2** recorded in MeOH

**Figure 17.** IR spectrum of compound **2**


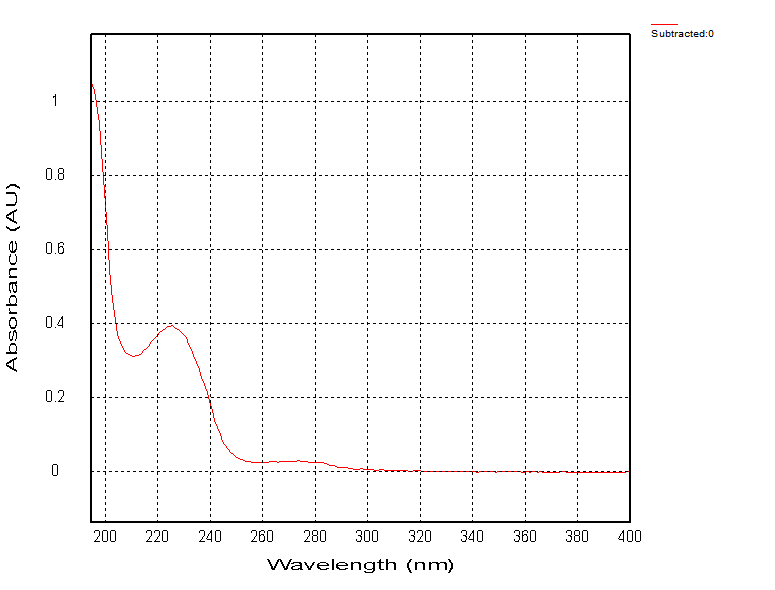


**Figure 18.** UV spectrum of compound **2**


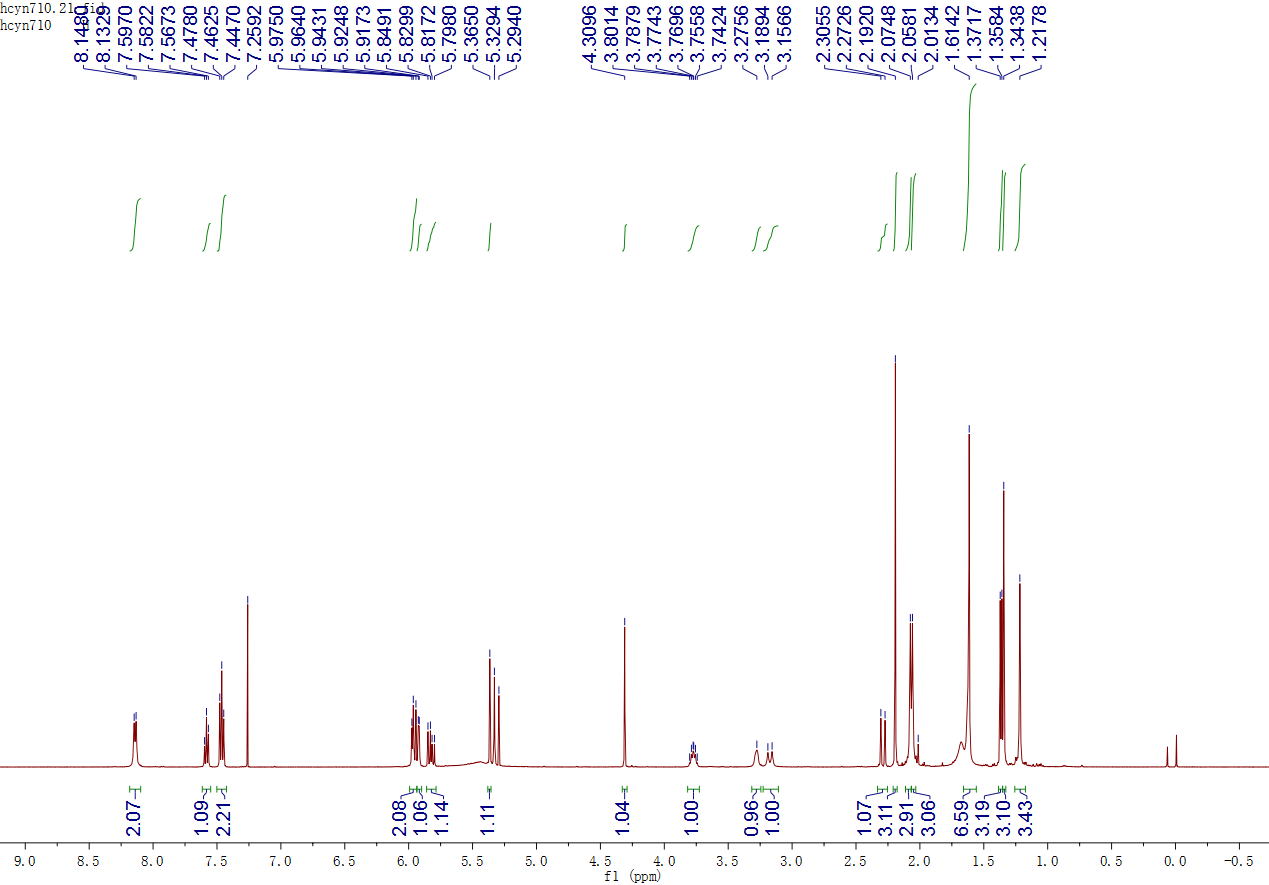


**Figure 19.**^1^H NMR spectrum of compound **3** recorded inCDCl_3_ at 500 MHz


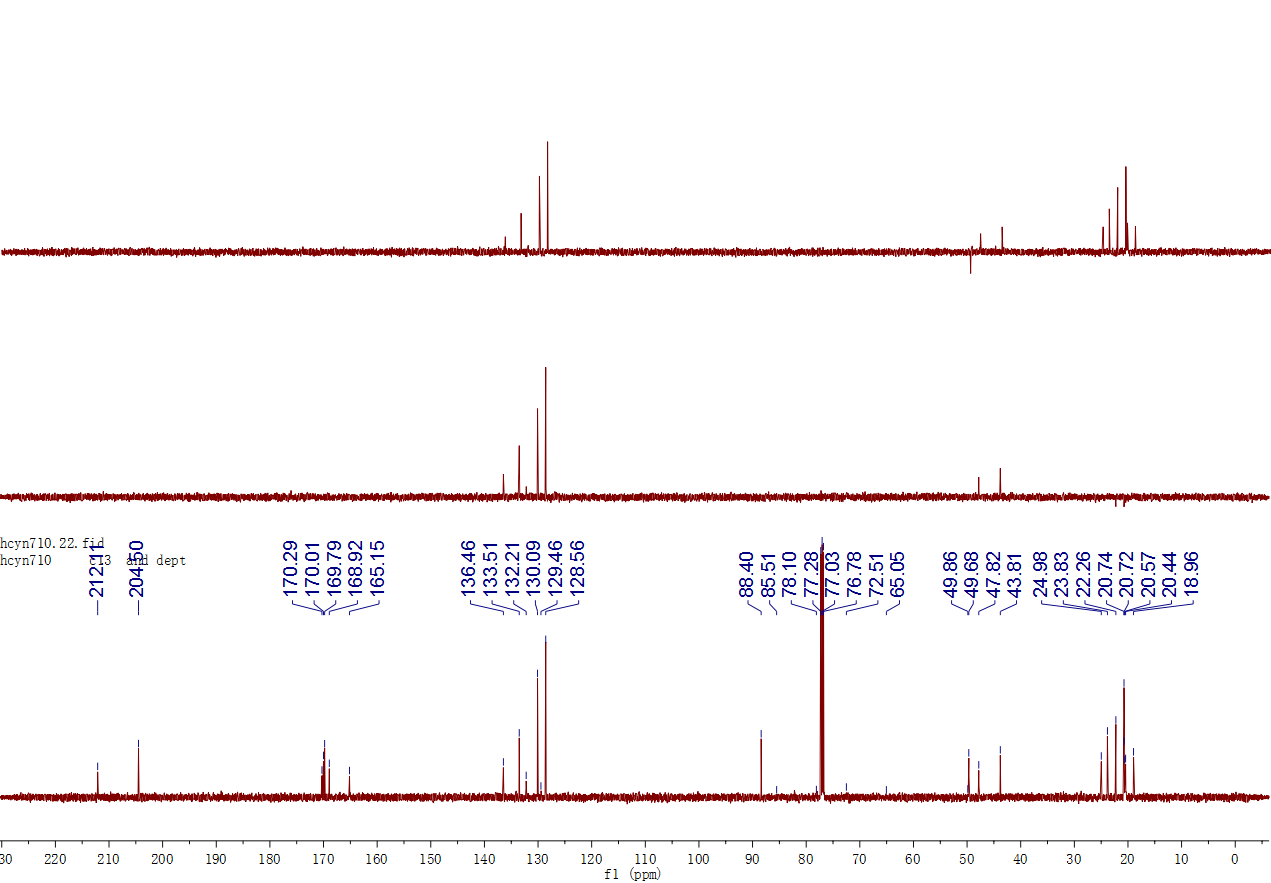


**Figure 20.**^13^C NMR spectrum of compound **3** recorded inCDCl_3_ at 500 MHz


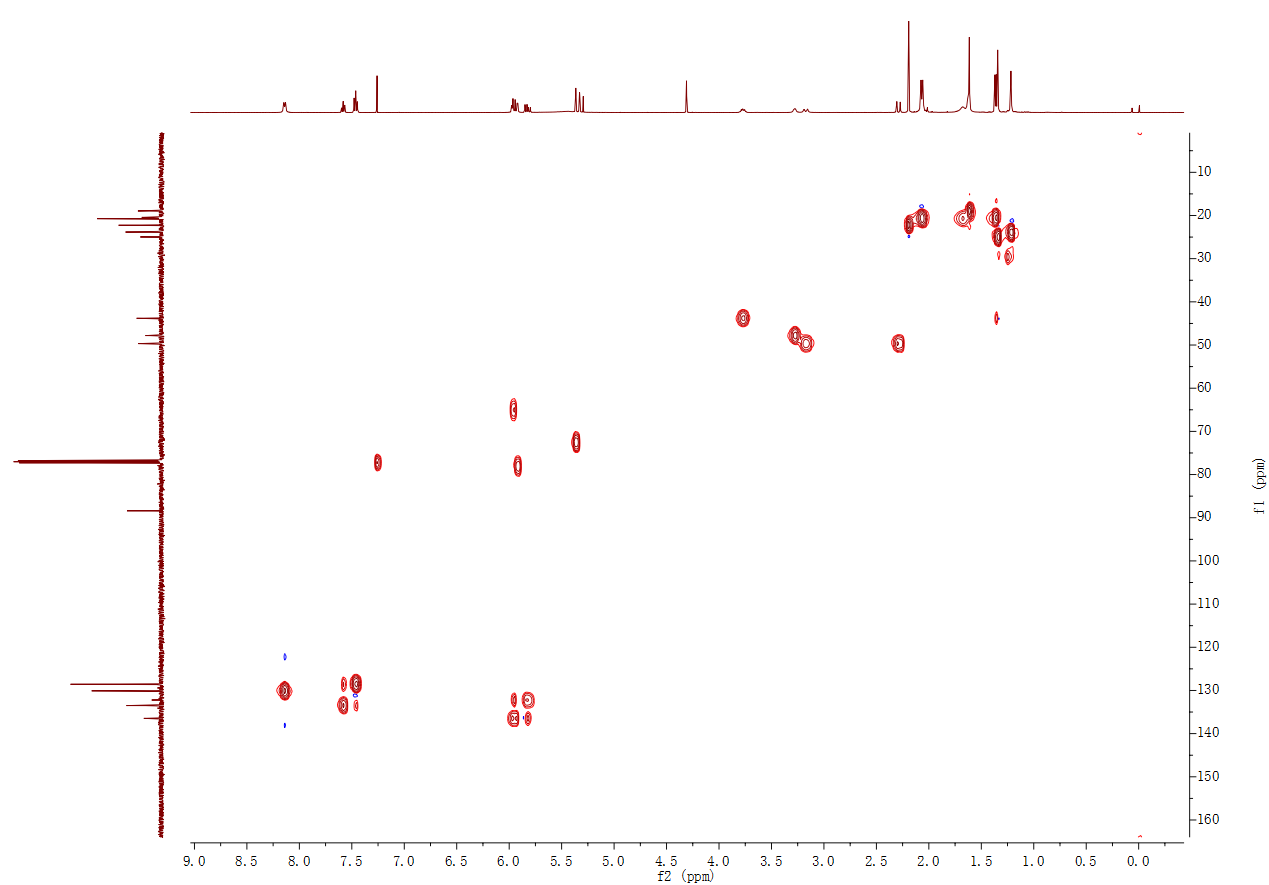


**Figure 21.** HSQC spectrum of compound **3** recorded in CDCl_3_


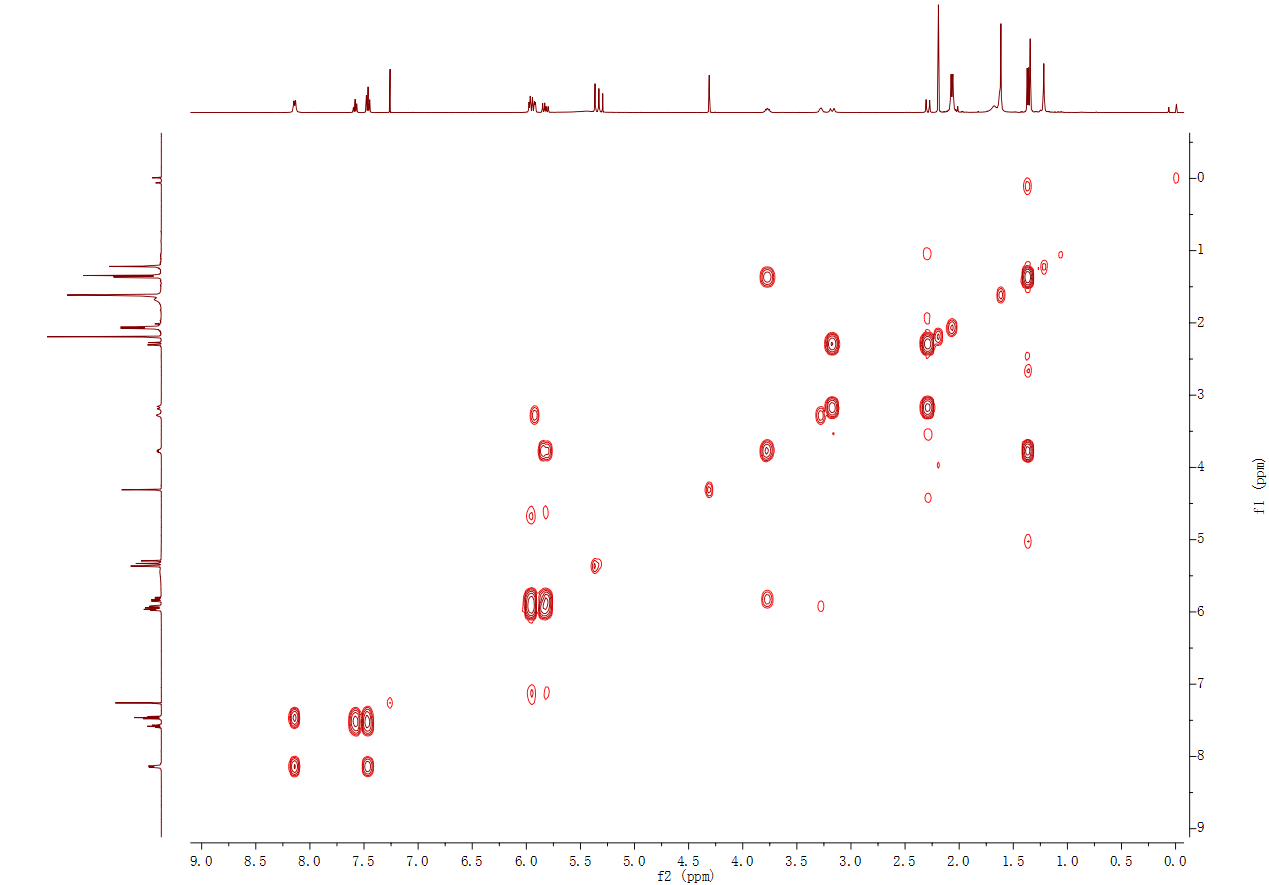


**Figure 22.** ^1^H-^1^H COSY spectrum of compound **3** recorded in CDCl_3_


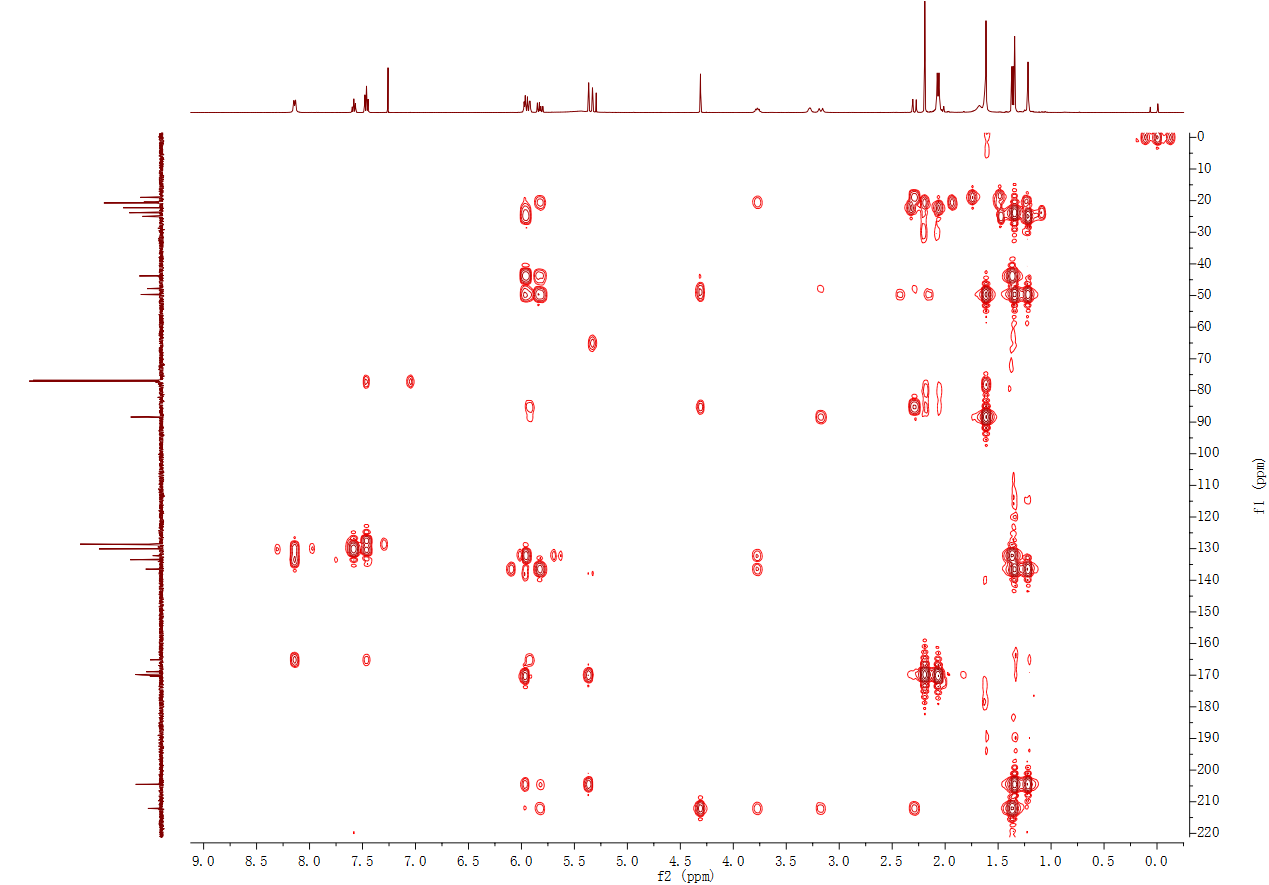


**Figure 23.** HMBC spectrum of compound **3** recorded in CDCl_3_


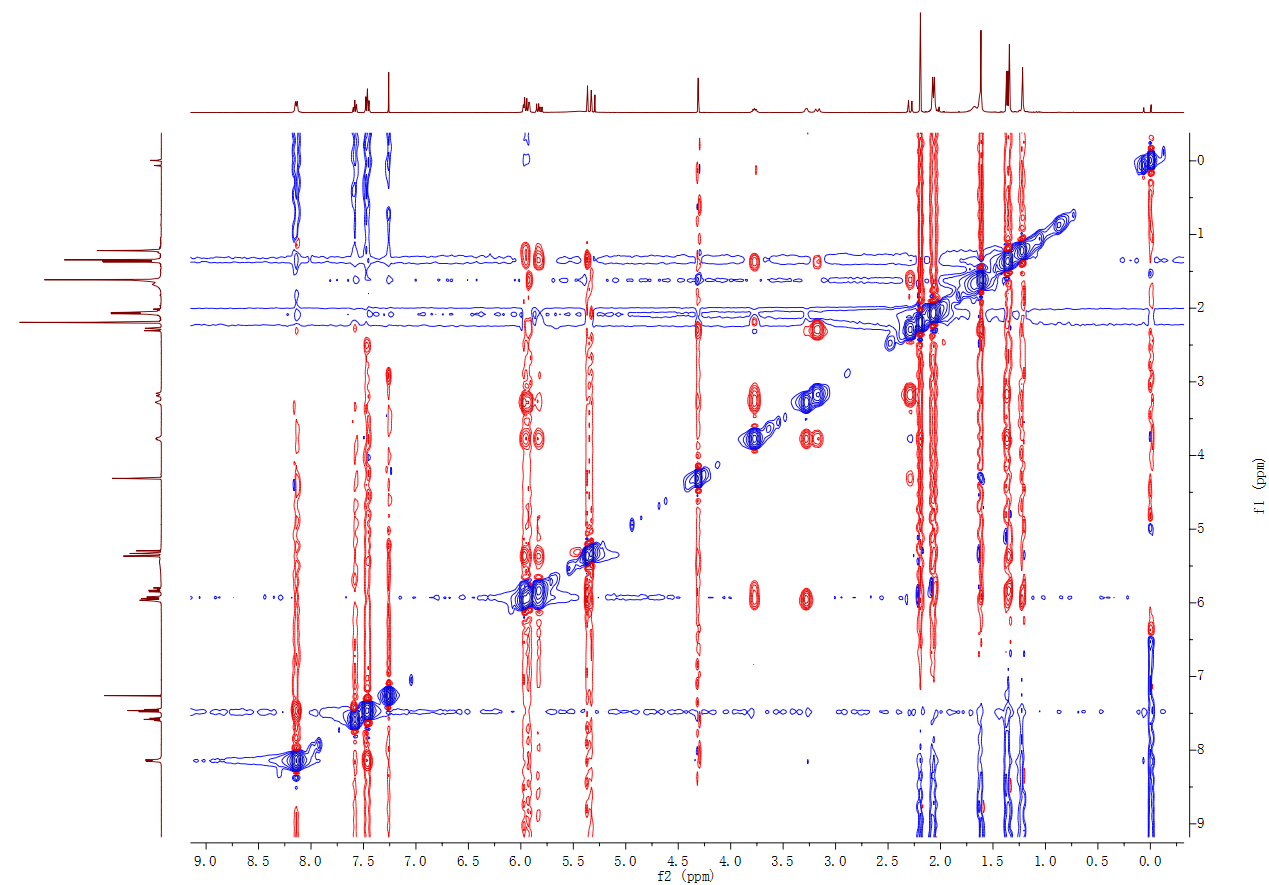


**Figure 24.** ROESY spectrum of compound **3** recorded in CDCl_3_


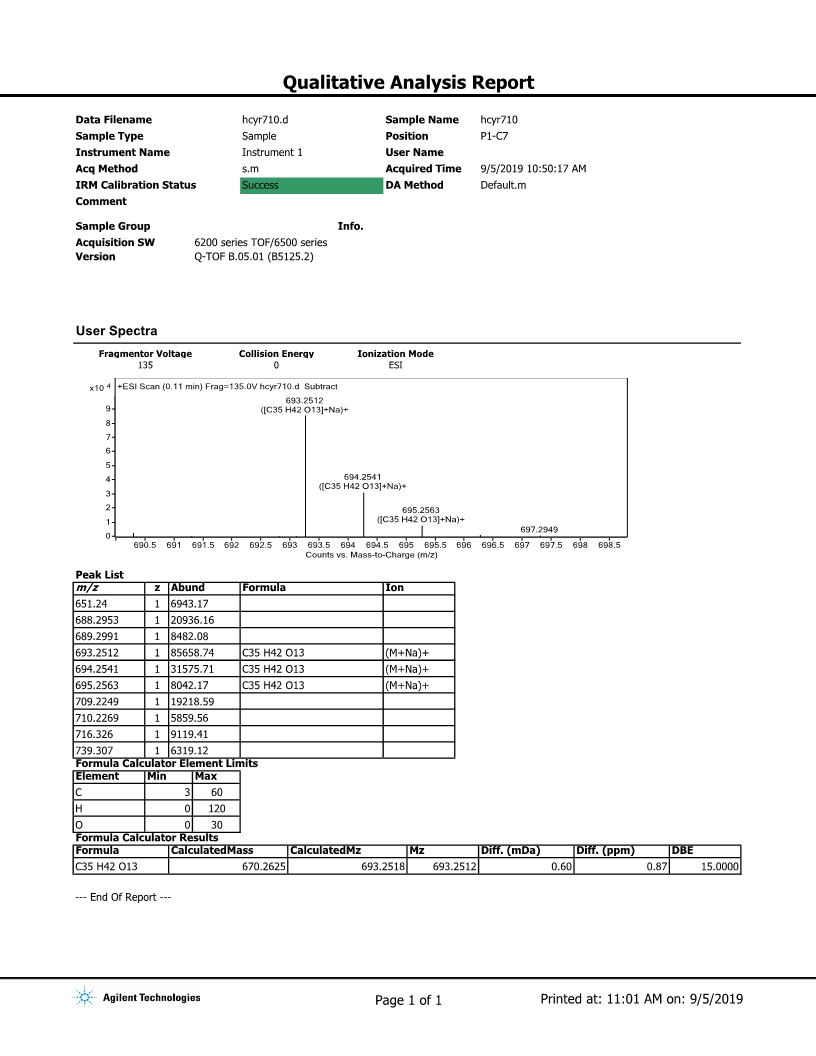


**Figure 25.** HRESIMS spectrum of compound **3** recorded in MeOH

**Figure 26.** IR spectrum of compound **3**


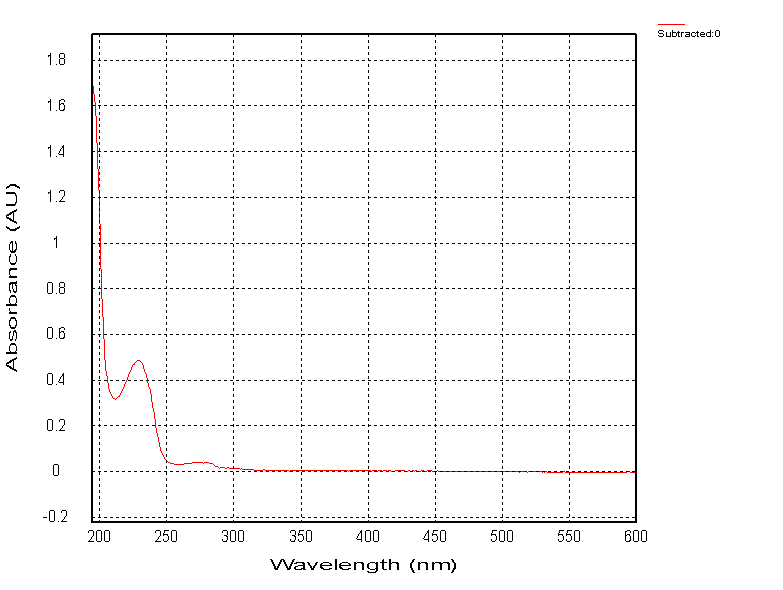


**Figure 27.** UV spectrum of compound **3**


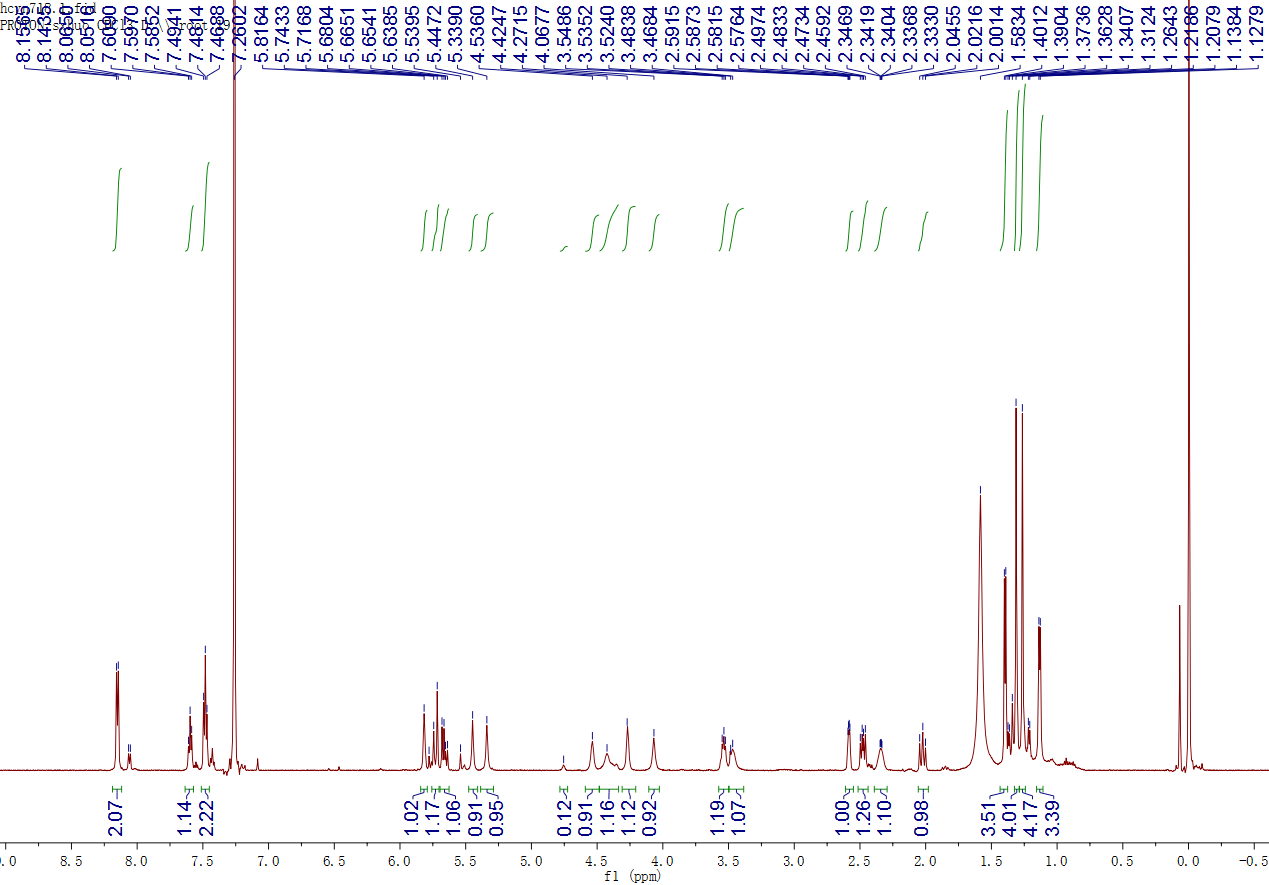


**Figure 28.**^1^H NMR spectrum of compound **4** recorded in CDCl_3_ at 500 MHz


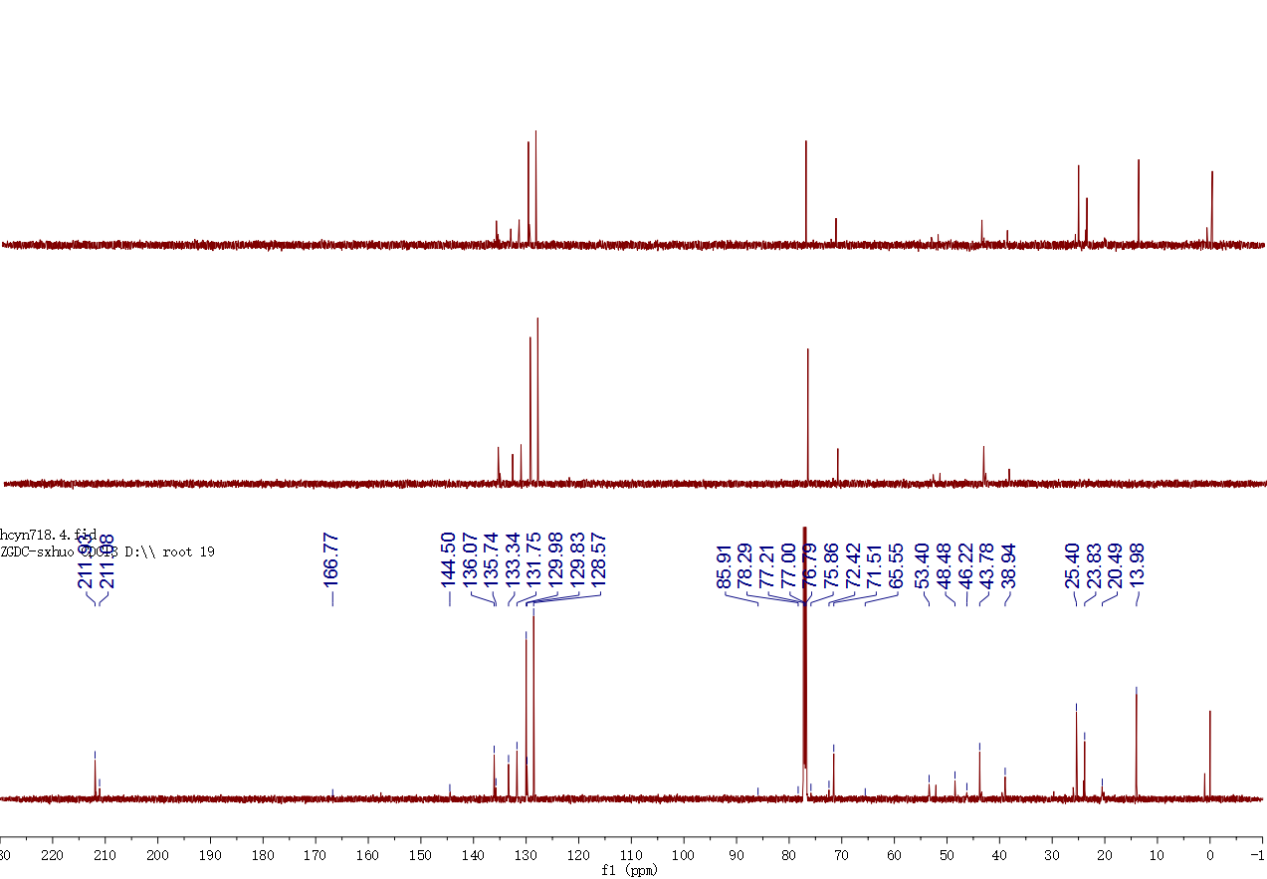


**Figure 29.**^13^C NMR spectrum of compound **4** recorded in CDCl_3_ at 500 MHz


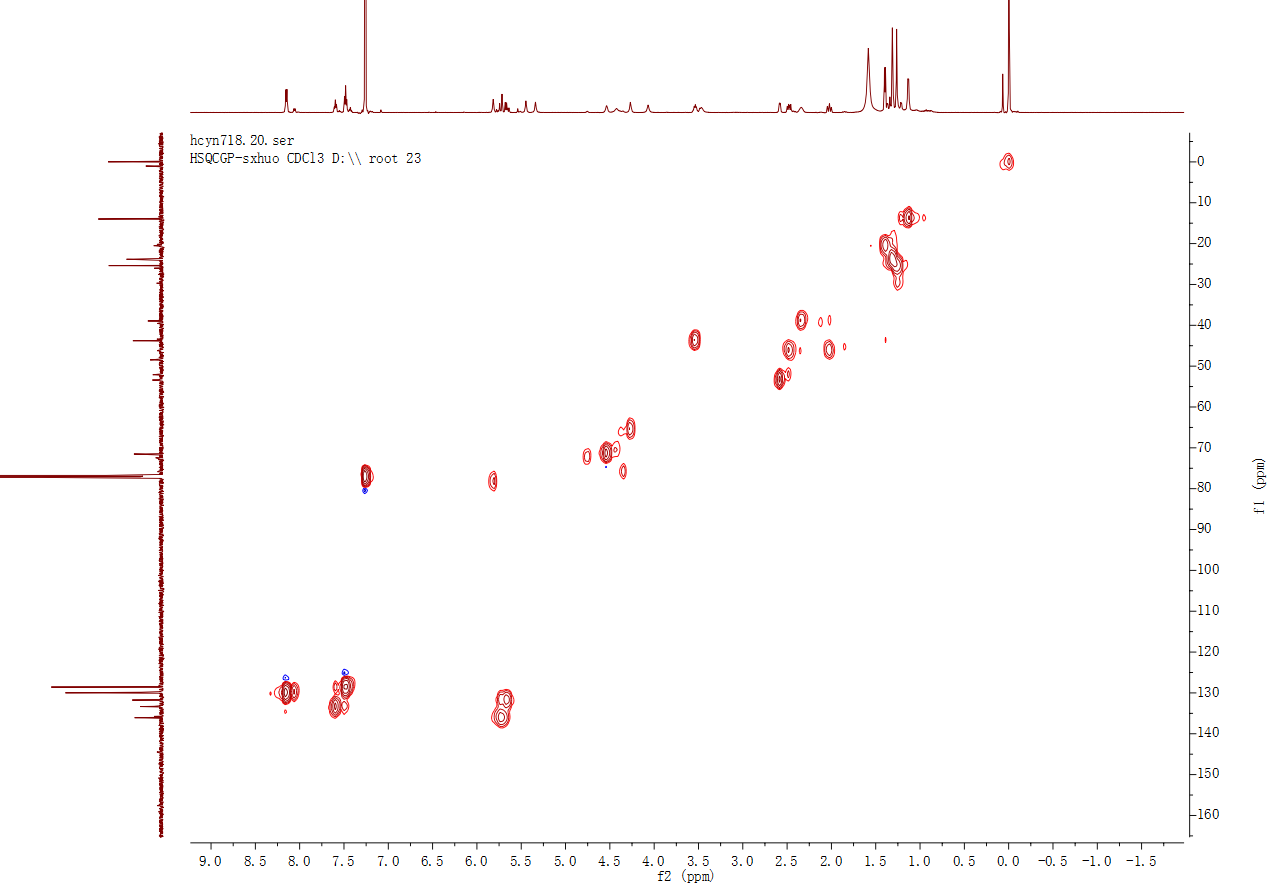


**Figure 30.** HSQC spectrum of compound **4** recorded in CDCl_3_


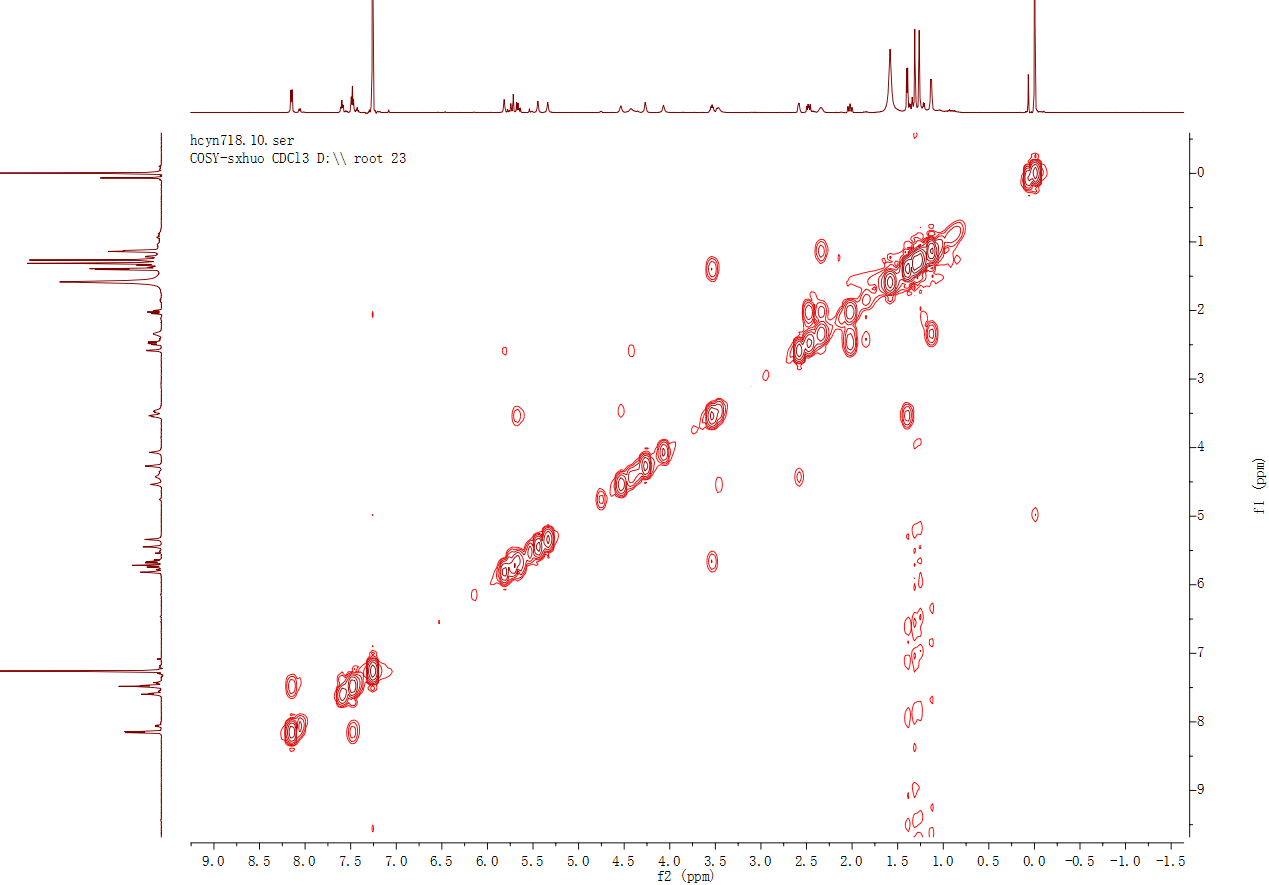


**Figure 31.** ^1^H-^1^H COSY spectrum of compound **4** recorded in CDCl_3_


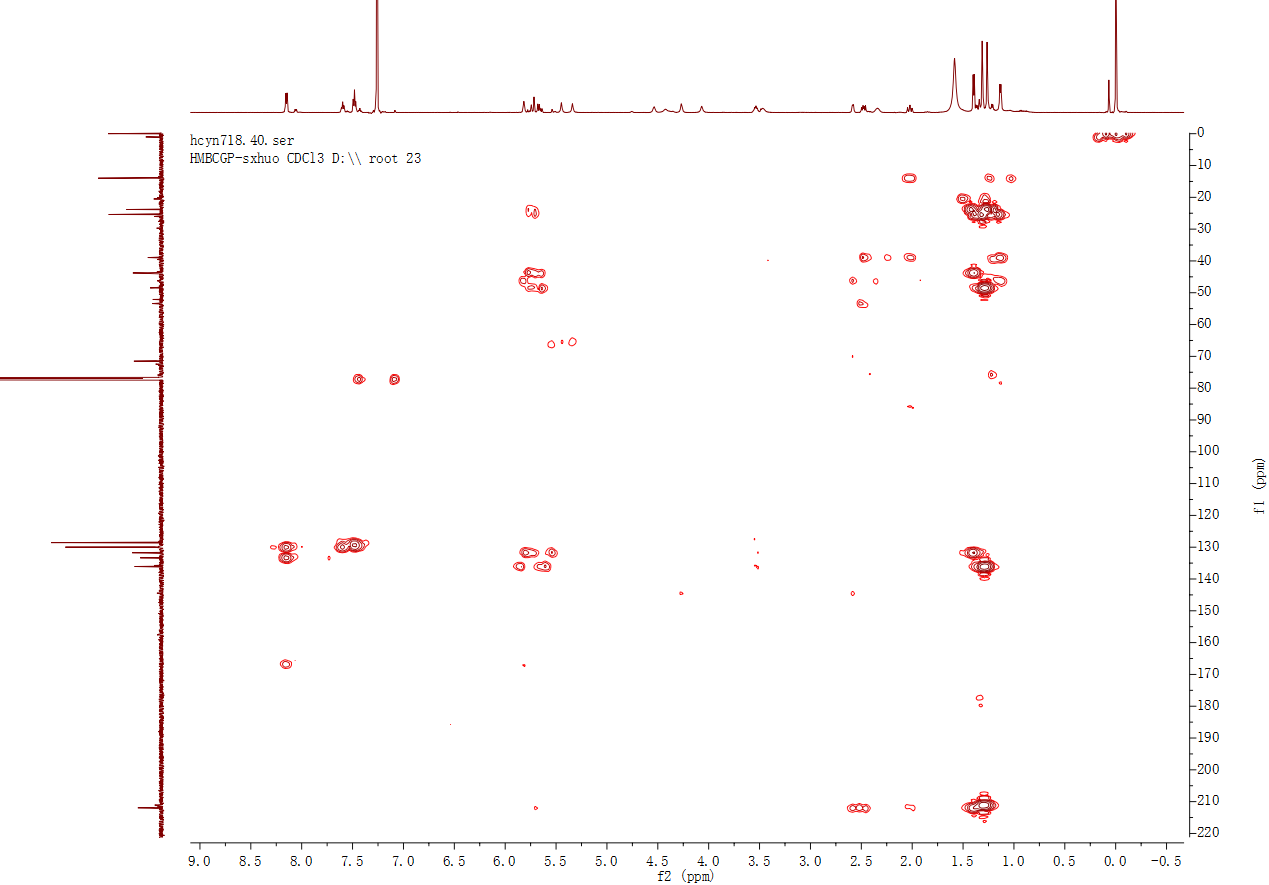


**Figure 32.** HMBC spectrum of compound **4** recorded in CDCl_3_


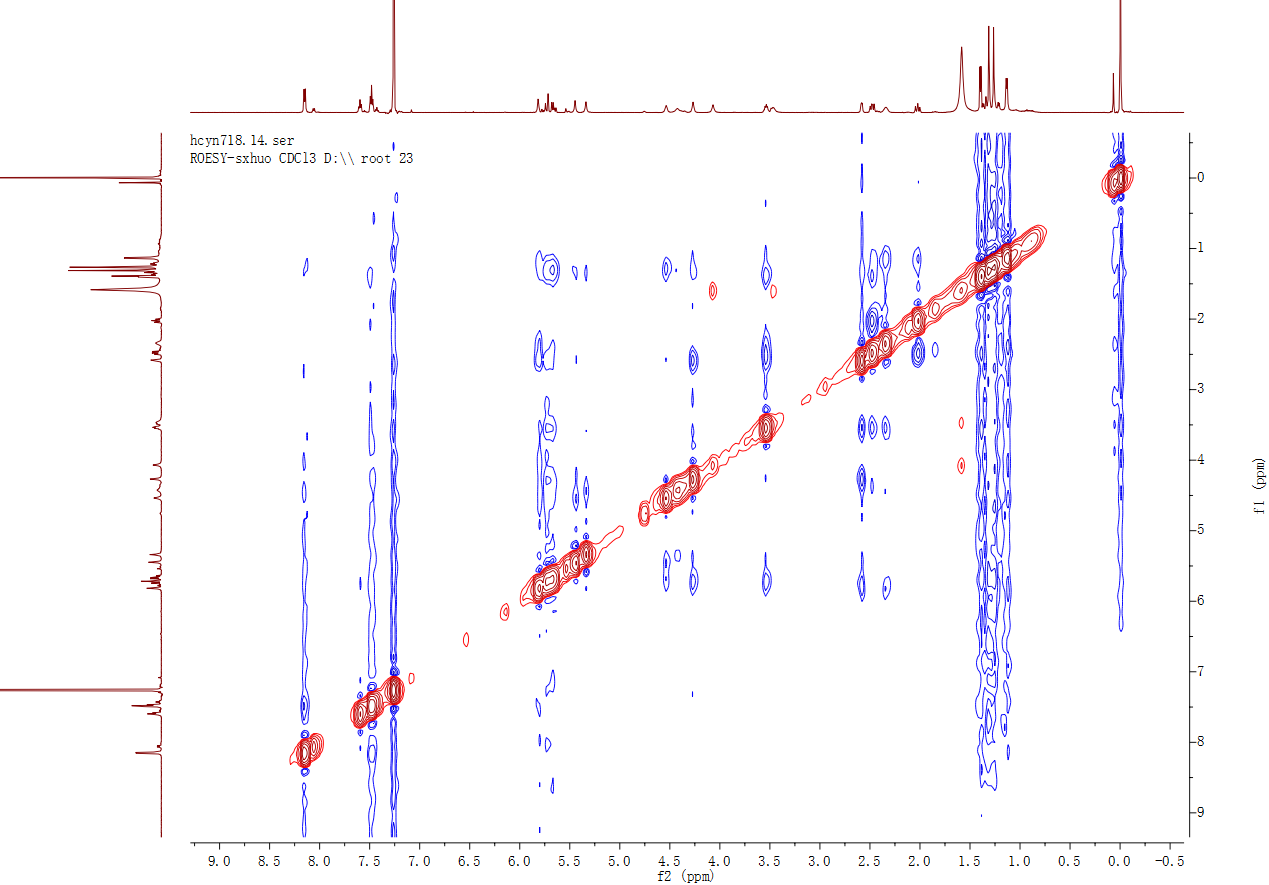


**Figure 33.** ROESY spectrum of compound **4** recorded in CDCl_3_


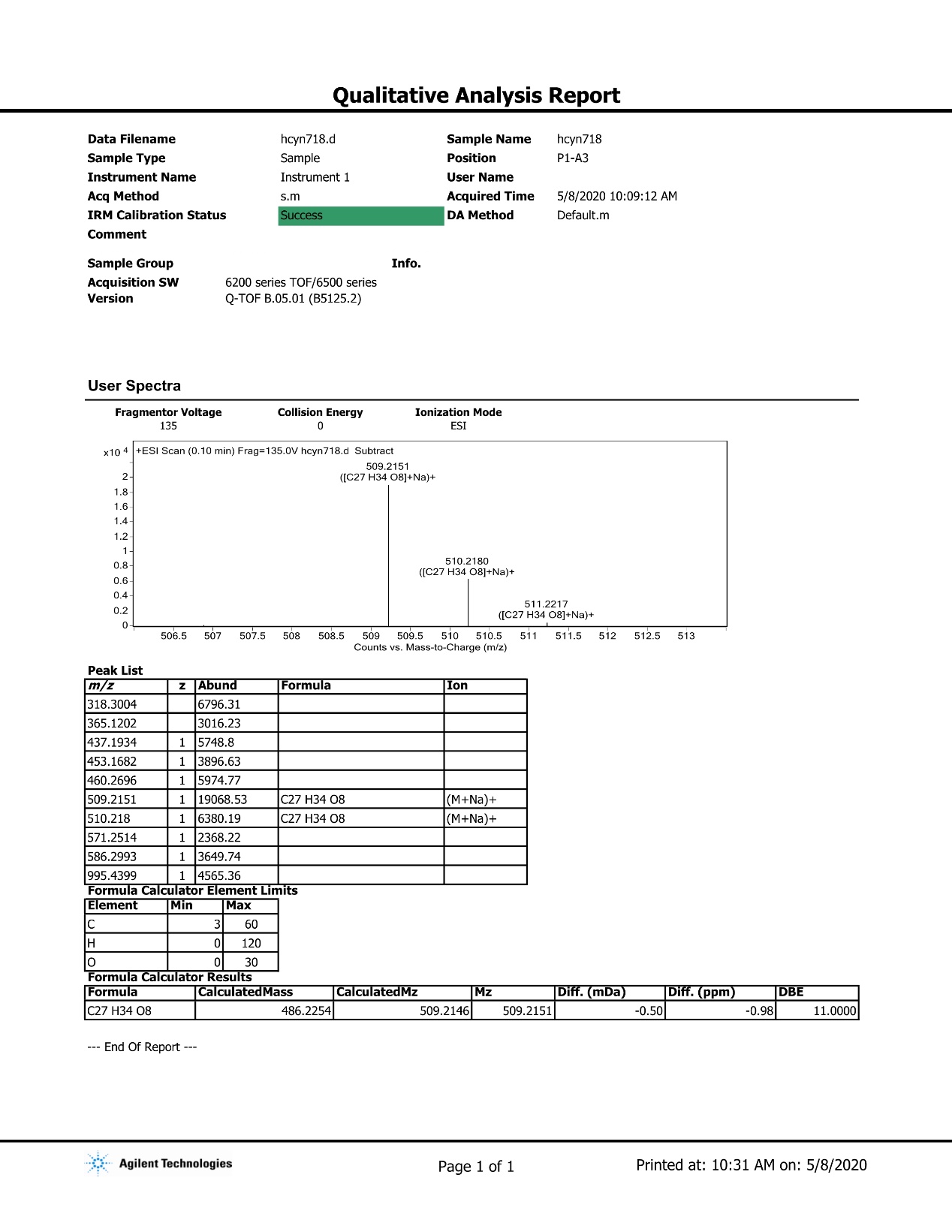


**Figure 34.** HRESIMS spectrum of compound **4** recorded in MeOH

**Figure 35.** IR spectrum of compound **4**


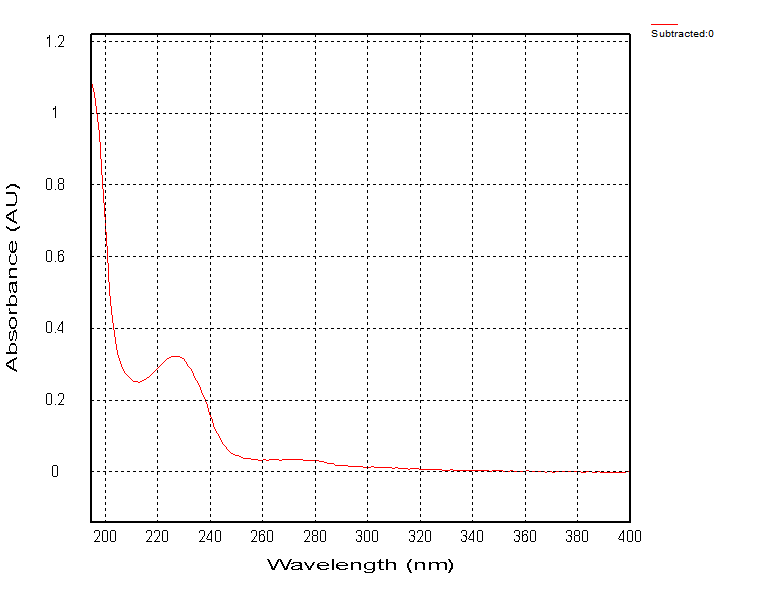


**Figure 36.** UV spectrum of compound **4**

**Table 1.** Crystal data and structure refinement for compound **1**

Identification code global

Empirical formula C_33_ H_40_ O_12_

Formula weight 628.65

Temperature 100(2) K

Wavelength 1.54178 Å

Crystal system Monoclinic

Space group P2**_1_**

Unit cell dimensions a = 10.0011(3) Å α= 90°.

b = 17.0114(4) Å β= 114.3100(10)°.

c = 10.2133(3) Å γ = 90°.

Volume 1583.54(8) Å3

Z 2

Density (calculated) 1.318 Mg/m3

Absorption coefficient 0.839 mm-1

F(000) 668

Crystal size 0.630 x 0.450 x 0.200 mm3

Theta range for data collection 4.751 to 70.249°.

Index ranges -12<=h<=12, -20<=k<=20, -12<=l<=11

Reflections collected 17488

Independent reflections 5524 [R(int) = 0.0390]

Completeness to theta = 67.679° 99.7 %

Absorption correction Semi-empirical from equivalents

Refinement method Full-matrix least-squares on F2

Data / restraints / parameters 5524 / 1 / 419

Goodness-of-fit on F2 1.065

Final R indices [I>2sigma(I)] R1 = 0.0330, wR2 = 0.0852

R indices (all data) R1 = 0.0330, wR2 = 0.0853

Absolute structure parameter 0.04(4)

Extinction coefficient n/a

Largest diff. peak and hole 0.178 and -0.269 e.Å-3
